# METRIN-KG: A knowledge graph integrating plant metabolites, traits, and biotic interactions

**DOI:** 10.1093/gigascience/giag051

**Published:** 2026-04-29

**Authors:** Disha Tandon, Tarcisio Mendes De Farias, Pierre-Marie Allard, Emmanuel Defossez

**Affiliations:** Faculty of Science, Institute of Biology, University of Neuchâtel, Rue Emile-Argand 11, 2000 Neuchâtel, Switzerland; SIB Swiss Institute of Bioinformatics, Amphipôle, Quartier UNIL-Sorge, 1015 Lausanne, Switzerland; SIB Swiss Institute of Bioinformatics, Amphipôle, Quartier UNIL-Sorge, 1015 Lausanne, Switzerland; Faculty of Science and Medicine, Department of Biology, University of Fribourg, Chemin du Musée 10, 1700 Fribourg, Switzerland; Faculty of Science, Institute of Biology, University of Neuchâtel, Rue Emile-Argand 11, 2000 Neuchâtel, Switzerland

**Keywords:** knowledge graphs, plant metabolomes, plant traits, biotic interaction

## Abstract

**Background:**

In recent years, biodiversity data management has emerged as a critical pillar in global conservation efforts. Today, the ability to efficiently collect, structure, and analyze biodiversity data is central to breakthroughs in conservation, drug development, disease monitoring, ecological forecasting, and agri-tech innovation. However, due to the vastness and heterogeneity of biodiversity data, it is often confined to databases for specific research areas in isolated formats and disconnected from other relevant resources. Crucial components of such data in the kingdom Plantae comprise metabolomes, with the vast array of compounds produced by plants; traits, which are measurable characteristics of plants that influence their growth, survival, and reproduction and affect ecosystem processes; and biotic interactions, including relationships of plants with other living organisms, affecting the ecosystem functions.

**Results:**

In this work, we present METRIN-KG (MEtabolomes, TRaits, and INteractions-Knowledge Graph), a powerful data resource simplifying the integration of diverse and heterogeneous data resources such as plant metabolomes, traits, and biotic interactions.

**Conclusions:**

The proposed knowledge graph provides an interface to interactively search for data relating to plant metabolomes, traits, and interactions. This, in turn, will facilitate the development of research questions in life sciences. In this context, we provide representative case studies on how to frame queries that can be used to search for relevant data in the knowledge graph.

## Introduction

### Interplay of metabolomes, traits, and biotic interactions

All species of our planet are connected by means of shared resources for sustenance, response to environmental effects, and multilevel biotic and abiotic interactions. An in-depth understanding of this multiscale network of interactions enables researchers from natural sciences, chemistry, microbiology, ecology, plant biology, and climate change to address critical questions in ecology, biodiversity conservation, agriculture, and human health. As referenced by studies before [1–3], there are 7 main shortfalls of biodiversity knowledge. Among these, Raunkiæran and Eltonian shortfalls emphasize the *lack of connected knowledge on species’ traits and interactions, as well as their corresponding relation to ecological functions*. The chemistry of life, by governing biotic interactions, resource access, environmental adaptation, and individual phenotype, provides a link to building the mechanistic network required to understand species’ processes underlying ecosystem functioning [4].

The metabolome, a key component of the chemistry of life, refers to all metabolites forming the substrates or products of enzymatic reactions in an organism. To a certain extent, the metabolome provides a bridge between the ecosystem functioning and the contextual information of species’ states across multiple scales (spatial, temporal, and environmental) [5–7]. In the Plantae kingdom, the sheer magnitude of metabolite diversity produced by plants is estimated to be between 1.5 and 25.7 million collectively for 400,000 plants [8], which poses significant challenges for metabolomic data analysis [9, 10].

It has been shown that metabolomes can provide a proxy for the estimation of the plant functions, thus revealing the cause-and-effect relationship between external environmental factors and plant fitness [9]. However, exploring the metabolome structure across spatial, temporal, and environmental scales requires extensive studies to unravel the interplay of hundreds of compound classes and their potential biological and ecological functions.

Since experimental approaches alone cannot provide such comprehensive datasets, aggregating data across multiple studies that combine metabolome with physiological or environmental data appears to be a necessary solution. Such information can include functional data like traits and codependence data like interactions.

Plant ecology focuses on how an organism’s traits influence its interactions with and in response to environmental conditions throughout its life cycle. Traits like plant height, seed mass, leaf area, leaf carbon, nitrogen, and phosphorus contents have been used to define plant processes across taxonomic (tree of life) [11–13], spatial (e.g., subalpine regions) [14], environmental (e.g., weather changes) [15], and temporal (e.g., reproduction during the life of plant or life history) scales [15–17]. However, the mechanistic understanding behind the effect of traits on plant fitness and functioning is not clear [18–21] because of the unexplained or ambiguous relation of traits and variation in ecosystem functioning.

Plant biotic interactions, including relationships with other plants, fungi, bacteria, and soil organisms, have been shown to influence ecosystem structure and function, ultimately shaping biodiversity patterns [22–24]. As sessile organisms, plant biotic interactions also rely heavily on chemical mediation, involving both volatile and nonvolatile compounds to compensate for their lack of mobility. Such interactions, particularly those involving insects, such as defense mechanisms or pollinator attraction, have been closely linked to the diversity of plant metabolites through evolutionary processes [25–29].

### Challenges in integrating high-dimensional data

In recent years, efforts have been made to include chemistry into the functional traits framework to resolve plant functions in ecosystems [30, 31]. Many studies have tried to combine the traits with specific compounds or classes of compounds [30–34], biotic interactions with traits [35–38], and biotic interactions with metabolomes [39–42]. Some studies have combined the three to decipher ecosystem functioning. For instance, they have examined plant traits, their interactions with soil biota, and related chemodiversity [43, 44]. Some have explored the effect of insect herbivory on plant traits and their secondary metabolite concentrations [45–47]. Others have investigated links between plant root traits, nutrient foraging, chemodiversity, and their symbiotic relationship with mycorrhizal fungi [48–50]. Additional research has focused on allelopathic interactions and nutrient mobilization during intercropping in agriculture [51, 52]. Studies also cover plant root traits, exudates (metabolites produced by roots), and their interactions with the rhizosphere microbiome [53]. Further examples include links between biotic interactions, elevation gradients, and metabolomics [54]; climate-induced plant host shift in insects [55]; plant microbe interactions and chemical defense mechanisms [56]; and insect herbivory and defense mechanisms facilitated by secondary metabolites [57–59]. Moreover, several reviews published during the past decade have stressed the importance of combining plant traits, interactions, and metabolomes [33, 60–67]. The main challenge in studies that combine these 3 components is the high dimensionality of each, coupled with limited resources available to characterize such complexity, which poses analytical limitations (e.g., incomplete spectral libraries, detection limits), suboptimal data availability, and cross-links across the 3 components.

The experimental approaches commonly used in functional ecology are powerful tools for exploring and describing specific mechanisms. However, they remain limited when it comes to disentangling complex processes operating at larger scales. Linking chemical pathways, compound classes, or molecular structures to ecological functions remains a major challenge in ecological research. Recent developments in databases and advances in data science offer a new potential to reach this goal. A data-driven approach is required to first map the existing knowledge in all 3 areas, followed by the development of research hypotheses. Building on the few previous studies in this direction, we explored various datasets and databases related to traits, interactions, and metabolomics, which ultimately inspired the core idea of this study.

Much information on the heterogeneity of metabolites biosynthesized by organisms lies locked in isolated tabular formats, Excel sheets, and PDF documents (as discussed in [68]). Few databases provide comprehensive information on metabolites or natural products [68–72]. The Earth Metabolome Initiative (EMI) [73] and its pilot project, Digital Botanical Gardens Initiative (DBGI) [74], were launched in 2022. They aim to document metabolic content for all known species on Earth (the initiating point being botanical gardens’ living collections), following the Findable, Accessible, Interoperable, Reusable (FAIR) guidelines [75]. Under the EMI umbrella comes the Experimental Natural Products Knowledge Graph (ENPKG) [69], a published resource of metabolomes from 1,600 tropical plant extracts. ENPKG uses a sample-centric approach comprising semantic annotations to structure large, heterogeneous metabolomics datasets into knowledge graphs. It also enables the harmonization of experimental data with publicly available resources. However, no ecological metadata were integrated in ENPKG.

Like metabolite data, multispecies-level interaction data also lie locked as pairwise correlation metrics in peer-reviewed research papers. While Global Biotic Interactions (GloBI) [76] and tools like BiotXplorer [77] have succeeded in collecting this information in a FAIR resource [78], they allow only partial multispecies-level interaction mapping and are limited to pairwise interactions. Such documentation of interactions is remarkable, yet it lacks complete multispecies-level interactions. Furthermore, higher-order interaction structures (e.g., multitrophic or trait-mediated interactions) are often analyzed post hoc using correlation-based summaries that abstract away mechanistic or phenotypic information. Similarly, data on plant trait heterogeneity are limited to a few resources like TRY [79, 80] and the global plant trait network [81].

There are individual databases listing organism traits [80, 82], metabolite–pathway relations of a limited number of plant species [83] as well as microbes [84, 85], pairwise interaction maps [76], medicinal herbs [86], and food [87]. However, no single resource available combines knowledge across organisms, detailing their traits, interactions, and complete/partial metabolomes, all of which are important for the in-depth understanding of the complex network of life.

### MEtabolomes, TRaits, and INteractions–Knowledge Graph

In this Data Note, we present the first efforts in combining publicly available data on plant traits, interactions, and metabolome from peer-reviewed research and databases in MEtabolomes, TRaits, and INteractions–Knowledge Graph (METRIN-KG). We have linked enriched metabolome datasets behind ENPKG [69] with high-dimensional data on plant traits from the TRY database [80], pairwise interaction data from GloBI [76], and annotated data on natural products from LOTUS [68] (available through Wikidata). To ensure semantic interoperability, we have used ontologies for knowledge representation like the Earth Metabolome Initiative Ontology (EMI Ontology) [88, 89] and the ENPKG ontology for natural product-specific concepts [69]. We used Ontop [90–92] and the Python rdflib library [93] to materialize the Resource Description Framework (RDF) triples. We further implemented a SPARQL Protocol and RDF Query Language (SPARQL) editor to query METRIN-KG and retrieve results.

We anticipate METRIN-KG to be useful for interactively searching related information on traits, interactions, and metabolome, thus guiding and inspiring the development of future research questions in the fields of ecology, biodiversity conservation, agriculture, and human health. Moreover, we believe this information could also be valuable to a wider audience beyond researchers, such as policymakers and public health professionals, by enhancing their understanding of the broader implications of research within their respective fields.

In the following sections, we provide details on how the datasets used in METRIN-KG were retrieved, structured, and linked. We also provide a brief overview of the construction of the EMI Ontology, discuss potential reuse of METRIN-KG, and present representative queries for exploring the knowledge graph.

## Methods

### Data retrieval from the TRY database

For this study, the pilot dataset was retrieved from the TRY website [94] by requesting data for 41 traits based on the most used functional trait categories in plant physiology studies [30]—plant height, seed mass, leaf area, leaf carbon content, leaf nitrogen content, leaf phosphorus content, stem specific density, leaf lifespan, leaf respiration rate, and photosynthesis rate (Table 1). Scientific names of the species from this dataset were mapped to Wikidata, as listed in the section “Taxonomy mapping.” The retrieved data are archived publicly in the file “TRYdb_40340.txt.gz” in a Zenodo repository [95].

**Table 1:** List of traits used for retrieving data from TRY database.

Trait identifier in TRY	Trait name
1	Leaf area
3108	Leaf area (in case of compound leaves: leaf, petiole excluded)
3109	Leaf area (in case of compound leaves: leaflet, petiole excluded)
3110	Leaf area (in case of compound leaves: leaf, petiole included)
3111	Leaf area (in case of compound leaves: leaflet, petiole included)
3112	Leaf area (in case of compound leaves: leaf, undefined if petiole is included or excluded)
3113	Leaf area (in case of compound leaves: leaflet, undefined if petiole is included or excluded)
3114	Leaf area (in case of compound leaves: undefined if leaf or leaflet, undefined if petiole is included or excluded)
11	Leaf area per leaf dry mass (specific leaf area, SLA or 1/LMA)
3115	Leaf area per leaf dry mass (specific leaf area, SLA or 1/LMA): petiole excluded
3116	Leaf area per leaf dry mass (specific leaf area, SLA or 1/LMA): petiole included
3117	Leaf area per leaf dry mass (specific leaf area, SLA or 1/LMA): undefined if petiole is included or excluded
3085	Leaf area per leaf dry mass (specific leaf area, SLA or 1/LMA) of leaf lamina
3086	Leaf area per leaf dry mass (specific leaf area, SLA or 1/LMA) petiole, rhachis, and midrib excluded
3106	Plant height vegetative
3107	Plant height generative
26	Seed dry mass
3660	Seed fresh mass
3836	Seed mass per fruit
4	Stem specific density (SSD, stem dry mass per stem fresh volume) or wood density
3452	Stem specific density (SSD, stem dry mass per stem fresh volume) or wood density: heartwood
3764	Stem specific density (SSD, stem dry mass per stem fresh volume) or wood density: stem
13	Leaf carbon (C) content per leaf dry mass
14	Leaf nitrogen (N) content per leaf dry mass
15	Leaf phosphorus (P) content per leaf dry mass
12	Leaf lifespan (longevity)
663	Leaf respiration rate in light per dry mass
512	Leaf respiration rate in light per leaf area
664	Leaf respiration rate in light per leaf respiration rate in the dark
2943	Leaf respiration rate in the dark as a fraction of photosynthetic carboxylation capacity (Vcmax)
665	Leaf respiration rate in the dark minus respiration in light, mass based
54	Leaf respiration rate in the dark per leaf area
41	Leaf respiration rate in the dark per leaf dry mass
500	Leaf respiration rate in the dark per leaf nitrogen (N) content
272	Leaf respiration rate in the dark temperature dependence
53	Photosynthesis rate per leaf area
974	Photosynthesis rate per leaf area: transition to TPU limited photosynthesis
40	Photosynthesis rate per leaf dry mass
135	Photosynthesis rate per leaf nitrogen (N) content (photosynthetic nitrogen use efficiency: PNUE)
134	Photosynthesis rate per leaf transpiration (photosynthetic water use efficiency: WUE)
3128	Photosynthesis rate per stomatal conductance

LMA: leaf dry mass per area; PNUE: photosynthetic nitrogen use efficiency; SLA: specific leaf area; SSD: stem specific density; TPU: triose phosphate utilisation; WUE: water use efficiency.

### Data retrieval from GloBI

The compressed stable release of interaction data was downloaded from the Zenodo archive of GloBI, version 0.8 from January 2025 [96], representing “species interactions tabulated as pairwise interactions in a zipped tab-separated values format” (as described on the GloBI website [97]). The creators of GloBI mention that “included taxonomic names are not interpreted, but included as documented in their sources.” The taxonomic identifiers and names were mapped to Wikidata as listed in the section “Taxonomy mapping.” The data are available publicly in the file “verbatim-interactions.tsv.gz” in the GloBI Zenodo repository [96].

### Taxonomy mapping

GloBI collates interaction information from standardized data from numerous sources, including online repositories and projects (e.g., Encyclopedia of Life), as well as data entered by individual projects through GloBI’s dataset-template [98]. The full list of data sources is available on its website [99]. The combination of these 2 approaches resulted in taxonomic identifiers from around 15 taxonomy databases (Supplementary Table S1) to be present in GloBI. The initial taxonomic mappings from these 15 resources, as well as their scientific names to Wikidata identifiers, were done using QLever’s [100] SPARQL user interface (UI) for Wikidata [101]. SPARQL queries used for mapping are as follows:

- Query [102] for mapping Wikidata identifiers to those from 15 taxonomy databases (Supplementary Table S1)
- Query [103] for retrieving Wikidata identifiers and their lineage

The full queries are listed in Supplementary data.

The retrieved data are available publicly in “wdTax” files at the METRIN-KG Zenodo repository [104].

For the TRY database, the scientific names were directly matched to those obtained from Wikidata to obtain corresponding Wikidata identifiers.

### Metadata mapping

GloBI provides limited mappings of life stages and body parts to Uber-Anatomy Ontology (UBERON) [105], Plant Ontology (PO) [106], Environment Ontology (ENVO) [107], Gene Ontology (GO; body parts only) [108], and Phenotype and Trait Ontology (PATO; body parts only) [109] for organisms involved in interactions (Supplementary Table S2). For biological sex, the names are provided in GloBI, but no specific mapping to existing ontologies.

With such limited mappings, it was crucial to map the raw text in metadata columns: body part, life stage, and biological sex. Such unmapped text were not single standardized terms; rather, it contained compound expressions, abbreviations, counts, symbols, or multiple entities within a single field. For example, body part entries included values such as “2 guts,” “abdominal cavity,” “abo,” and “abomasum/si.” Biological sex fields contained symbolic or aggregated values (e.g., “+,” “-,” “?”), numeric summaries (e.g., “10 M,9F,” “11 males, 4 females, 2 juveniles”), or multilingual expressions (e.g., “11 machos | 9 hembras”). Life stage fields similarly contained compound or inconsistent representations, such as “adult; adult; egg,” “adult; larvae,” “0, I, II, III,” or mixed capitalization and punctuation.

As part of the metadata mapping process, such entries were first parsed using delimiters and normalization rules to separate them into individual candidate terms, which were then mapped independently to ontology entities.

To semantically match candidate terms with ontology concepts, we developed a script using ontology parsing and sentence embeddings. The code including the parsing and semantic matching [110] is publicly available in the METRIN-KG GitHub repository. We utilized the Python Owlready2 library version 0.47 [111] to load a suite of biomedical and environmental ontologies, namely, those originally present in GloBI, as well as others from their Persistent Uniform Resource Locators (PURL) (see Supplementary Table S2). For life stages and body parts, the full suite was considered, whereas for biological sex, only UBERON [105] and PATO [109] were considered.

From each ontology, we extracted class labels and associated synonyms (including exact, broad, and related synonyms where available) as candidate terms for matching. Synonyms were collected from Web Ontology Language (OWL) annotation properties such as “hasExactSynonym,” “hasBroadSynonym,” and “hasRelatedSynonym.” Each candidate term was paired with its primary label and class Internationalized Resource Identifier (IRI) for traceability.

To compute semantic similarity, we used the pretrained “all-MiniLM-L6-v2” model from the Python SentenceTransformers library version 3.3.1 [112]. This model is a lightweight transformer trained to generate 384-dimensional dense vector embeddings that reflect the semantic content of short text spans. It is particularly suited for tasks such as semantic textual similarity and clustering.

All ontology terms (labels and synonyms) were encoded into dense vector representations using this model. Similarly, each user-provided input term for unmapped life stages, body parts, and biological sex was independently embedded into the same vector space. Term embeddings were computed using the model’s “encode()” method with “convert_to_tensor=True,” which produced PyTorch-compatible tensors suitable for high-performance vector operations.

Cosine similarity was computed between each input term vector and all ontology term vectors using the “util.pytorch_cos_sim” function in SentenceTransformers. For each input term, the ontology term with the highest cosine similarity score was identified as the top candidate match. Cosine similarity is a common metric for comparing the orientation, rather than the magnitude, of 2 vectors. Given 2 vectors A and B, cosine similarity is defined as


\begin{eqnarray*}
{\mathrm{cosine}}\_{\mathrm{similarity}}\left( {{\mathrm{A}},{\mathrm{B}}} \right) = {\mathrm{A}}.{\mathrm{B}}/\left| {\mathrm{A}} \right|.\left| {\mathrm{B}} \right|
\end{eqnarray*}

Values range from −1 to 1, where 1 indicates identical direction (i.e., maximum similarity) and 0 indicates orthogonality (no similarity). For each input term, we computed its similarity against all ontology terms. The term with the highest similarity score was selected as the top candidate match. While all terms received a candidate match, only matches with a similarity score ≥0.7 were flagged for manual review. This threshold ensures that low-confidence suggestions are carefully examined, while very low-similarity terms are treated as unmatched.

All results were output to a comma-separated values (CSV) file, including the input term, matched ontology label, its primary label, the ontology class IRI, and the similarity score.

While the automated process provided high-quality initial suggestions, all matches were manually reviewed and corrected to ensure semantic appropriateness. Corrections were informed by domain-specific knowledge and contextual relevance, particularly when terms had multiple meanings or high similarity scores did not guarantee ontological alignment. This 2-stage process, automated semantic matching followed by manual curation, ensured both scalability and accuracy in aligning nonstandardized input terms with controlled ontology concepts.

The above workflow was also run for matching the units of measure for trait data from TRY to the units’ vocabulary [113] provided by the Quantities, Units, Dimensions, and Types (QUDT 2.1 schema) [114], followed by manual correction.

The mapped data are archived in the folders “globi” and “trydb” under processed data in the METRIN-KG Zenodo repository [104].

### EMI ontology

**Figure 1:**
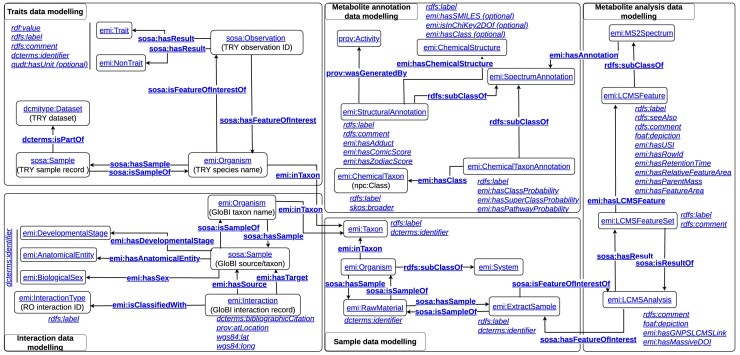
Snapshot of the data schema of the Earth Metabolome Initiative (EMI) ontology. Classes are represented in round-edged rectangles. Entity relationships are represented by directional arrows.

EMI [73] is a global effort to profile the metabolic content of all currently known species on our planet. Here, knowledge representation plays a key role in correctly capturing, contextualizing, and structuring the vast amount of chemical diversity data that have been and will be generated in the upcoming years. Consequently, this will facilitate data (re)use and interoperability. To accurately represent the EMI knowledge, we explored several general-purpose and domain-specific ontologies to design a framework to describe chemical compounds (e.g., natural products) and their related data such as geolocation, provenance, organism sample metadata, and organism interactions. The EMI ontology reuses and repurposes, where applicable, several other ontologies beyond the life sciences such as World Wide Web Consortium’s (W3C) [115] Sensor, Observation, Sample, and Actuator (SOSA) ontology [116], which was primarily designed for other applications, including the Open Geospatial Consortium (OGC) use cases. OGC is a consortium that aims to improve access to geospatial and location information [117]. Furthermore, semantic reconciliation powered by biocuration is at the core of our proposed ontology. For example, by applying the EMI ontology version 1.0, we can accommodate and harmonize different organismal, chemical, and material sample taxonomies as well as vocabularies, such as the Relation Ontology (RO) [118], to define interactions between organisms (e.g., “has pathogen”). The other ontologies reused in EMI are those of the Simple Knowledge Organization System (SKOS) [119], standard units of measures from QUDT [114], geo-locations from the basic geo (World Geodetic System 1984 [WGS84] lat/long) vocabulary [120], and natural product-specific concepts from the ENPKG ontology [121]. Moreover, in addition to making several existing ontologies interoperable for the EMI knowledge representation, we created more than 100 new concepts and relations that can be easily identified since they start with the EMI ontology prefix emi: [88], for example, the term *emi:NonTrait*, a nontrait structured value. All new and reused terms that constitute the EMI ontology are documented and available online [88]. The code and a tutorial to build the metabolite-specific knowledge graph component of METRIN-KG with this ontology are available in the EMI Ontology GitHub repository [122]. Figure 1 shows the EMI ontology schema.

### Knowledge graph construction

**Figure 2:**
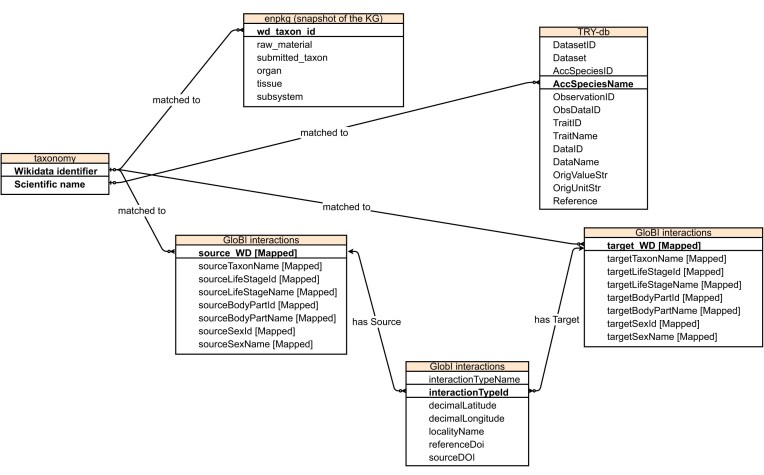
Schema of relations between different entities of integrated datasets of metabolome, traits, and interactions. Entities mapped are represented in bold, and the corresponding relationship is represented by directional arrows.

Based on the EMI ontology as a data schema (Fig. 1), we built a knowledge graph (KG) that semantically enriches, integrates, and interoperates 3 data sources—enriched metabolome datasets behind the ENPKG, trait data from TRY-db, and interaction data from GloBI. We developed the KG by combining subgraphs in 2 stages:

- Ontop tool [90–92] was applied for developing the subgraph from enriched datasets (metabolite annotation, molecular networks, and taxonomical resolution results) originally used to build the ENPKG graph [123, 124]. Ontop is a virtual knowledge graph system where SPARQL queries are translated into Structured Query Language (SQL) queries based on the predefined mappings. Ontop also provides means to materialize a KG according to these mappings. Therefore, to build the graph, 1 table for each tabular file was created in a relational database with a simplistic data schema. The files were loaded in this database through mysql version 8.2 [125]. Finally, to construct the KG, mappings were defined between the relational schema and the EMI ontology using the Ontop mapping language [90]. With these mappings [126], the EMI ontology [88], and the relational database, the RDF triples were materialized with Ontop to compose the KG.
- For developing the subgraphs from the TRY database and GloBI, Python rdflib library version 7.0.0 [93, 127] was used. To construct them, the tab-separated value (TSV) files obtained for each of these 2 resources were used. These tables were then connected through Wikidata identifiers (Fig. 2) for taxonomy whenever they were available, as indicated in section “Taxonomy mapping.” Mappings were defined between the relational schema and the EMI ontology using rdflib’s inherent capability to map ontology elements.
  The process for incorporating mappings to metadata for GloBI (source/target taxonomy, life stage renamed to developmental stage, body part renamed to anatomical entity, and biological sex) and TRY database (taxonomy and trait’s units of measure) was performed before generating the RDF triples.
  The code to develop the subgraphs is available in the METRIN-KG GitHub repository [128].

The RDF files can be downloaded from the METRIN-KG Zenodo repository [104] in the folder “KG” under processed data.

### Indexing the knowledge graph and implementing the SPARQL endpoint

We used Qlever [100], a SPARQL engine, to index our knowledge graph by implementing the steps described in qlever-control [129]. A SPARQL editor to query the indexed graph is available at [130]. This endpoint can also be accessed through [131] for programmatic purposes. An example for the programmatic access is provided on METRIN-KG’s GitHub wiki [132]. The underlying operational endpoint is currently available at [133]. The stable URL [130] functions as a long-term and stable identifier to safeguard accessibility should the operational endpoint be modified in the future. This interface includes several examples covering specific use cases for the 3 datasets (ENPKG metabolites, TRY-db traits, and GloBI interactions). Some of these use cases are presented as case studies in the section “Data reuse and case studies.”

To provide the SPARQL editor user interface for the indexed graph, we implemented qlever-ui [134, 135] with a few changes. To improve the user experience when navigating entities in the knowledge graph, we extended the qlever-ui interface to support fallback representations for unavailable or missing webpages. This was achieved by incorporating SPARQL DESCRIBE queries into the frontend of the UI. The modified interface attempts to retrieve a minimal RDF-based summary of the requested entity when the original resource is not accessible (e.g., due to missing data in the knowledge graph). When such a condition is met, the interface automatically issues a SPARQL DESCRIBE query for the corresponding IRI. The result is rendered in a simplified RDF triple view, providing context about the entity based on available data in the backend knowledge base. The implementation is available via a fork of the official qlever-ui GitHub repository here [136].

## Data Description

### Mapping of TRY data

**Figure 3:**
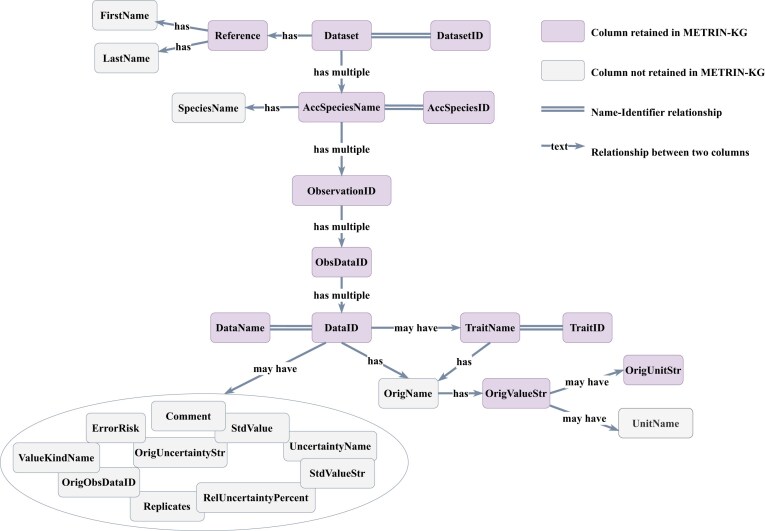
Original structure of the traits dataset retrieved from the TRY database. Purple-shaded rectangles represent columns retained for METRIN-KG. Gray-shaded rectangles represent columns not retained for METRIN-KG. Column relationships are represented by directional arrows.

The original structure of the TRY datasets is modeled as plant species mapping to trait and nontrait data, as well as related metadata like information on studies from which the data were obtained, as shown in Fig. 3. The column names depicted in the figure are taken directly from the CSV table retrieved from TRY, the description of which is provided in Supplementary Table S3. Columns retained in the knowledge graph are indicated in the table and the figure. Overall, 20,272,589 records for 70,748 unique plant species were retrieved in a TSV format (Supplementary Table S4).

The dataset was refined to a minimal form by removing columns that contained redundant or ambiguous information. For example, author first and last names were excluded, as this information is already available in the retained “Reference” field. Likewise, the “OriginalName” column duplicated data present in “DataName” and was therefore omitted. “Replicates” is also an entity mentioned in “DataName” and therefore not retained. The TRY database includes standardized values for certain traits in the columns “ErrorRisk,” “StdValue,” “StdValueStr,” “OrigUncertaintyStr,” “UncertaintyName,” “ValueKindName,” and “RelUncertaintyPercent.” However, these were excluded due to their limited coverage across the dataset. This selective retention helped minimize redundancy and ensured consistency within the constructed knowledge graph. The metrics of the dataset following this refinement are as follows:

- In total, 65,675 unique species names (“AccSpeciesName”; see Supplementary Table S4) out of 70,748 from the TRY database were mapped to Wikidata (see section “Taxonomy mapping”).
- Overall, 1,826,445 traits and 17,212,303 nontrait (e.g., number of replicates, latitude, longitude) records, data values, and corresponding units (Supplementary Table S4) were retained after mapping 65,672 species to Wikidata identifiers.

Query to retrieve metrics is described in [137].

### Mapping of GloBI data

**Figure 4:**
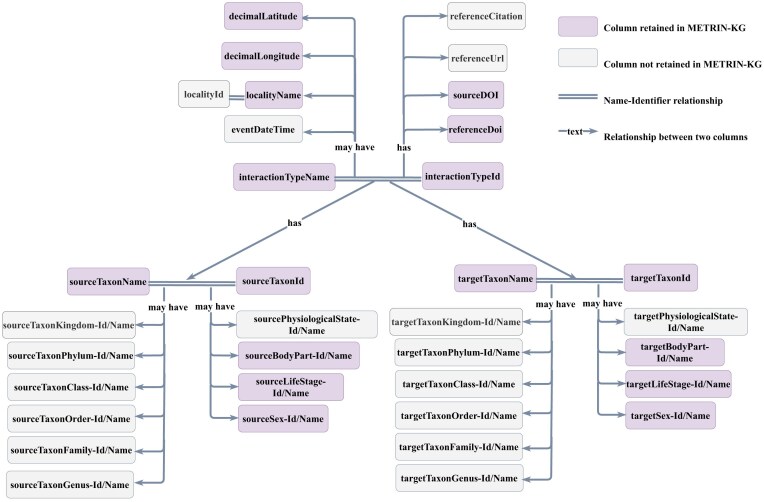
Original structure of the interactions dataset retrieved from GloBI. Purple-shaded rectangles represent columns retained for METRIN-KG. Gray-shaded rectangles represent columns not retained for METRIN-KG. Column relationships are represented by directional arrows.

The original structure and column descriptions of the GloBI data are described in Fig. 4 and Supplementary Table S5. The column descriptions are also provided on the GitHub account of GloBI as depicted on its website [138]. Columns retained in the knowledge graph are indicated in the table and the figure. Lineage information was not retained, as it can be retrieved via federated queries through Wikidata. Physiological stage data were omitted due to the absence of reliable mappings to established ontologies or controlled vocabularies. The “eventDateTime” field was removed owing to ambiguity—specifically, it was not clear whether it referred to the timing of the interaction event or the time of observation.

Overall, 20,480,925 records were obtained from 1,747,254 unique taxonomic identifiers and/or names (Supplementary Table S4), of which 609,087 were mapped to 337,293 unique Wikidata identifiers. Overall, 12,872,681 GloBI records for only the mapped taxonomic identifiers were retained.

Queries for the number of unique Wikidata identifiers and the number of records are provided in [139] and [140], respectively.

At the time of writing this manuscript, 1,867 body part names were mapped to 996 ontology terms, 621 life stage names were mapped to 227 ontology terms, and 57 biological sex names were mapped to 10 ontology terms.

### Bias

Biases in the mapped GloBI data in our knowledge graph reflect those documented for GloBI, including taxonomic and geographic skews, which are extensively discussed in the GloBI literature [76].

Metabolite coverage is primarily derived from 2 sources: (i) untargeted metabolomics data from approximately 1,600 plant extracts from the Pierre Fabre collection, as described in the ENPKG study [69], and (ii) compound–taxon associations curated via Wikidata/LOTUS, which are also known to be biased toward well-studied taxa and compounds [68].

## Data reuse and case studies

### Potential reuse and expansion of data

The current state of the knowledge graph provides a solid foundation. Its utility can be significantly enhanced by incorporating additional data sources and refining its structure.

#### Data reuse for studies in life sciences research

Researchers can query METRIN-KG to identify potential relationships between plant traits, interactions, and the presence or bioactivity of specific natural products. For example, one could query for plants with specific traits known to be associated with defense mechanisms and then explore the natural products they produce. The integrated data can be used to build predictive models for natural product occurrence or bioactivity based on plant traits and ecological context. This information can help prioritize plant species or natural product classes with a higher likelihood of possessing desired bioactivities. In addition, by analyzing trait–interaction–metabolome associations, researchers can prioritize plant species or ecological contexts that are more likely to yield novel or interesting natural products. Investigating such ecological roles of natural products can provide clues about their potential mechanisms of action and possible drug targets.

METRIN-KG can also facilitate the investigation of how natural products mediate species interactions. For instance, one could explore how specific compounds are associated with pollination or herbivory defense in plants with particular traits. The understanding of the evolutionary pressures that shape the diversity of natural products can be investigated by linking them with the respective host traits and interactions.

In the context of potential data reuse for studies in life sciences research, we aimed to retrieve data from METRIN-KG on a few known questions in plant ecology, agriculture, and biodiversity. Table 2 lists the questions and the respective links to the SPARQL queries.

**Table 2:** Examples of SPARQL queries for METRIN-KG and links to queries.

Query	Title	Subject	Direct link to queries	Snapshot GitHub commit link to queries
1 (CS1)	List interactions of all species that have an IUCN status of near threatened	Conservation science	https://kg.earthmetabolome.org/metrin/11 [141]	https://kg.earthmetabolome.org/metrin/11/v/53581b1 [142]
2 (CS1)	List traits of all species that have an IUCN status of near-threatened	Conservation science	https://kg.earthmetabolome.org/metrin/12 [143]	https://kg.earthmetabolome.org/metrin/12/v/53581b1 [144]
3 (CS1)	List all metabolites produced by species with near-threatened IUCN status and with values available (or greater than a specific value) for trait “seed dry mass”	Conservation science	https://kg.earthmetabolome.org/metrin/13 [145]	https://kg.earthmetabolome.org/metrin/13/v/53581b1 [146]
4 (CS2)	List traits (and their values) of plants producing Diterpenoids	Functional ecology	https://kg.earthmetabolome.org/metrin/14 [147]	https://kg.earthmetabolome.org/metrin/14/v/53581b1 [148]
5 (CS3)	Natural producers (and their interactions that might be useful in agriculture) of onopordopicrin (wd:Q27107580), which might exhibit antimicrobial and cytotoxic activities, especially against human-derived macrophages and against epidermoid carcinoma cells	Human health	https://kg.earthmetabolome.org/metrin/20 [149]	https://kg.earthmetabolome.org/metrin/20/v/53581b1 [150]
6 (CS4)	List links between host–parasite and host–allelopathic interactions, where host is an agricultural crop, and allelopath is usually a plant that inhibits growth of parasites, which are usually insects. Inhibition happens by root/stem/leaf exudates of the allelopath.	Sustainable agriculture	https://kg.earthmetabolome.org/metrin/18 [151]	https://kg.earthmetabolome.org/metrin/18/v/53581b1 [152]
7 (CS5)	Retrieve data of 4 traits in leaf economics spectrum	Theoretical ecology	https://kg.earthmetabolome.org/metrin/22 [153]	https://kg.earthmetabolome.org/metrin/22/v/53581b1 [154]
8 (CS5)	Retrieve metabolites of organisms with data available from 4 traits in leaf economics spectrum	Theoretical ecology	https://kg.earthmetabolome.org/metrin/23 [155]	https://kg.earthmetabolome.org/metrin/23/v/53581b1 [156]
9	List of possible natural locations of plants that produce senkyunolide, which is a thalide for cerebral disorders	Human health	https://kg.earthmetabolome.org/metrin/16 [157]	https://kg.earthmetabolome.org/metrin/16/v/53581b1 [158]
10	List metabolites of plants that interact with plant parasite moth *Orgyia postica*	Functional ecology	https://kg.earthmetabolome.org/metrin/17 [159]	https://kg.earthmetabolome.org/metrin/17/v/53581b1 [160]
11	A list of interactions depicting connections between parasitoids harmful for insects living as parasites on plants	Functional ecology	https://kg.earthmetabolome.org/metrin/19 [161]	https://kg.earthmetabolome.org/metrin/19/v/53581b1 [162]
12	List possible interactions of plants that can produce norhyoscyamine, a plant secondary metabolite	Functional ecology	https://kg.earthmetabolome.org/metrin/21 [163]	https://kg.earthmetabolome.org/metrin/21/v/53581b1 [164]

CS: case study; IUCN: International Union for Conservation of Nature.

#### User-friendly approach to add queries as examples within METRIN-KG

We devised a method for the users of METRIN-KG to propose a query and incorporate it within the examples (e.g., queries listed in Table 2) using the SPARQL query editor proposed in [165, 166]. Once a user proposes a query on EMI’s sparql-examples GitHub repository’s issues section, it will allow us to review it for inclusion in the examples. Once the review and corrections are complete, the query will be available on the SPARQL query endpoint [130]. While the examples listed in Table 2 provide several complex queries, this option allows users to provide context-specific queries useful for building research questions.

A tutorial for contributing queries to METRIN-KG is provided on its GitHub repository wiki [167].

#### Adding data or federating over knowledge graphs presenting datasets from other publicly available resources

Incorporating environmental data (e.g., climate data and soil data) could allow for the analysis of how environmental factors influence traits, interactions, and natural product production and distribution. Including data on the timing of biological events (e.g., flowering times) could add a temporal dimension to the analysis of interactions and natural product occurrence. In addition, incorporating image data (e.g., plant morphology) and spectral data (e.g., metabolomic profiles) could provide richer characterizations of the entities within the knowledge graph. Integrating broader ecological context, such as community composition and ecosystem dynamics, could provide a more holistic understanding of the relationships. Where ethically and legally appropriate, incorporating curated traditional knowledge related to plant uses and natural products could add valuable perspectives. Moreover, including data from other natural product databases (e.g., PubChem [70], ChEMBL [71, 168]) could broaden the coverage of chemical compounds and their properties.

#### Enhancing ontological structure and semantics

For developing more granular relationship types, moving beyond simple pairwise interactions to include more specific relationship types (e.g., pollination, herbivory, symbiosis) with associated properties (e.g., strength, specificity) could be useful. The first efforts in this direction are taken by GloBI [76] and BiotXplorer [77]. However, they are limited to pairwise interactions and not connected to other types of data, which our knowledge graph provides. A useful step in this direction could be to implement semantic reasoning, hence inferring new relationships and knowledge that are not explicitly stated in the data.

#### Democratizing METRIN-KG access: querying in natural language

In order to make available the knowledge in the METRIN-KG for non-SPARQL-savvy users, we applied ExpasyGPT [169], a large language model (LLM)–driven tool based on lightweight metadata that allows for querying knowledge graphs in natural language. This tool facilitates querying METRIN-KG as well as various other life science databases in plain English. In other words, ExpasyGPT aids in constructing SPARQL queries in response to users’ text-format questions. Therefore, the final response is not given by the LLM but by querying METRIN-KG, which contains curated and high-quality data, mitigating well-known LLM issues such as hallucinations, lack of domain-specific knowledge, and black-box behavior. Consequently, this makes answers verifiable and reproducible. More precisely, ExpasyGPT implements a context optimization approach that relies on 2 main sources of metadata: (i) pairs of question-query examples and (ii) an automatically generated description of used classes and predicates. Both (i) and (ii) should be defined using well-known W3C vocabularies such as Vocabulary of Interlinked Datasets (VoID) and Shapes Constraint Language (SHACL), accessible through the SPARQL endpoint. Applying ExpasyGPT is straightforward since the metadata already exist, as described in Table 2, and is automatically done. A tutorial on how to use ExpasyGPT is provided on the METRIN-KG GitHub repository wiki [170].

### Case studies

Out of the 12 examples listed in Table 2, we built 5 case study (CS) summaries for 8 as listed below.

#### CS1: List traits, interactions, and metabolites of all species that have an International Union for Conservation of Nature (IUCN) status of near-threatened

Subject—Conservation science

The queries are described in [141, 143, 145] (Table 2).

Some studies have addressed the need to identify the threat status of plants by studying their functional traits [171–173]. Moreover, 1 study has suggested approaches for the conservation of threatened plant species using functional trait patterns [174]. Some other studies have used predictive modeling to enhance knowledge of interacting species pairs (e.g., predator–prey interactions) [175, 176] in the context of their habitat and traits. In addition, some researchers have suggested and reviewed ecological restoration strategies for threatened ecosystems based on functional traits and plant–animal interactions [177–179].

Keeping such broad studies in mind, we developed 3 SPARQL queries retrieving the traits, interactions, and metabolites of plant species with an IUCN status of near-threatened. Such queries would aid the researchers in collecting data relevant to near-threatened plant species (in some cases, insects) and developing strategies to model patterns specific to climate change, thereby developing ecological restoration plans.

Basic metrics obtained from the results of the queries are provided in Table 3. The results obtained from the 3 queries contain 9,299 unique species with highly heterogeneous data coverage. While interaction data were available for most species (8,598), trait measurements were substantially sparser (1,051 species, 19,279 measurements across 98 traits), and metabolite data were limited to only 47 species (818 unique InChIKeys and 1,000 records). Thirty-seven species possessed all 3 data types.

**Table 3:** Metrics for CS1.

Metric	Value
Total unique species (all data)	9,299
Unique species with trait data	1,051
Unique species with metabolite data	47
Unique species with interaction data	8,598
Unique species with traits and metabolites	47
Unique species with all three data types	37
Total unique traits	98
Total unique metabolites (InChIKeys)	818
Total unique interaction types	24
Total trait measurements	19,279
Total metabolite records	1,000
Total interaction records	12,396

While these 3 queries can be combined into one, we kept them separate to improve efficiency and retain modularity.

#### CS2: List traits and their values for plants producing diterpenoids

Subject—Functional ecology

The query is described in [147] (Table 2).

Recent studies have utilized trait data to understand the implications of diterpenoids producing invasive plant species and their allelopathic relationships with agricultural crops [180–185]. In the context of agriculture, there have been reviews of plant traits and their effects on insect herbivory [186], crop resilience (e.g., rice) of plants producing diterpenoids [187–189], and the implications of drought stress on diterpenoid-producing plants and their traits [190]. In the context of biodiversity conservation, there have been studies on plant traits and their metabolic profile [191]. In addition, there has been research on plant interactions with microbial communities of plants where the leaf surface has been reported to contain diterpenoids (tobacco and phyllosphere) [192].

Considering such diverse research on diterpenoid-producing plants or where diterpenoids are known to be extracted from plant surfaces, we developed a simple query to list traits of plants that have been known to produce or harbor diterpenoids.

**Figure 5:**
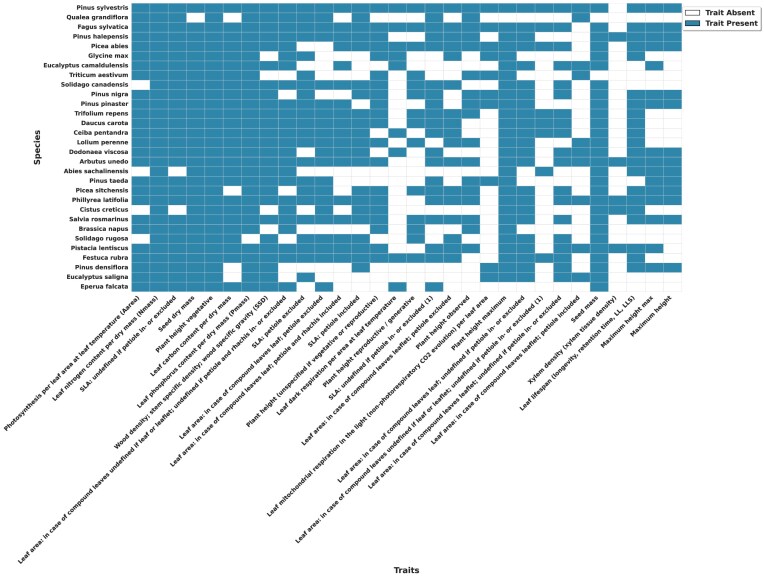
CS2–Trait coverage of the top 30 species selected based on measurement frequency from the results. Blue shows measured traits for a species; white shows no trait measured.

Figure 5 provides the trait coverage of top 30 species selected based on measurement frequency from the results. Supplementary Tables S6 and S7 provide complete summaries of species and trait distributions. The dataset contains 98 traits measured across 2,131 species, with photosynthesis per leaf area being the most frequently measured trait (16,472 measurements), followed by leaf nitrogen content (14,841 measurements) and specific leaf area (14,698 measurements). *Pinus sylvestris* has the highest number of trait measurements (14,557 records), followed by *Qualea grandiflora* (5,260 records) and *Fagus sylvatica* (4,978 records).

#### CS3: Natural producers and their biotic interactions that might be useful in the agriculture of onopordopicrin

Subject—Human health

The query is described in [149] (Table 2).

Onopordopicrin is a metabolite produced by several plants, such as *Arctium lappa* and the genus *Shangwua*. It has been shown to potentially exhibit antimicrobial and cytotoxic activities, especially against human-derived macrophages and against epidermoid carcinoma cells [193]. There is less scientific evidence to support these claims, and research in this area has only recently been enhanced [194–197], making onopordopicrin an appropriate and timely subject for prospective research studies in human health and pharmacology.

To specifically target the research in this area, we developed a query to retrieve the natural producers of onopordopicrin and their biotic interactions.

**Figure 6:**
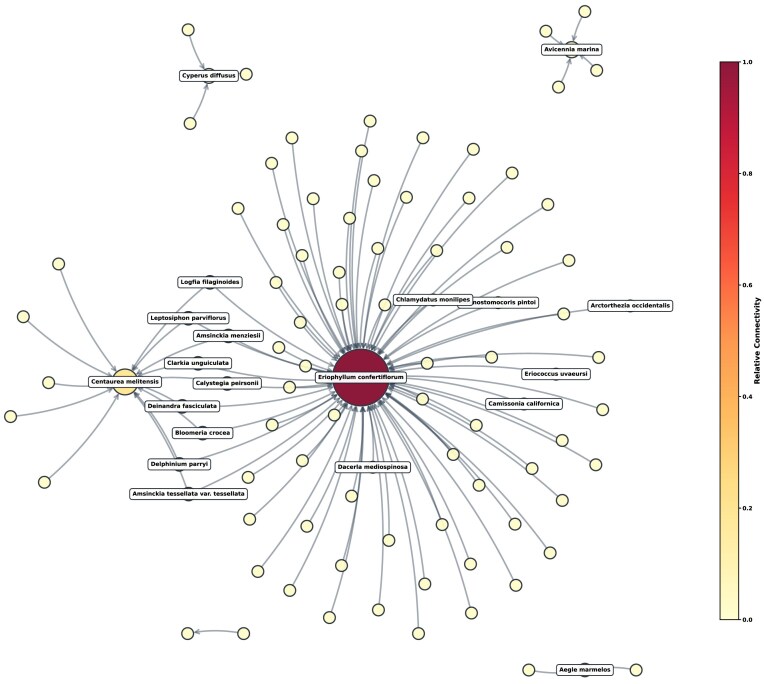
CS3–Interaction network for diterpenoid-producing plant species. Node size and color represent connection count. Only the top 20 species with the highest connection count are labeled.

The results comprise 98 diterpenoid-producing species connected through 101 biotic interactions. While we did not find *A. lappa* and genus *Shangwua* in the results, *Eriophyllum confertiflorum* emerges as a hub species with 77 incoming connections that interacts with numerous other organisms (Fig. 6). The network shows a highly skewed degree of distribution, where a small number of plant species (e.g., *Centaurea melitensis* with 14 connections) dominate interactions, while most species participate in only 1 or 2 interactions each.

#### CS4: Allelopathy interactions in push–pull agriculture

Subject—Sustainable agriculture

The query is described in [151] (Table 2).

Studies on allelopathy have advanced our knowledge of plant interactions with other organisms, particularly in agriculture [198–200] and invasion ecology [180]. For instance, species of the genus *Desmodium*, through their root and stem exudates, may protect crops of maize (*Zea mays*) and sorghum (*Sorghum*) against attack from stemborer arthropods and the weed *Striga hermonthica* [201, 202]. Besides focused research [203], there are few studies exploring the allelopathic properties and the plant metabolites responsible for them.

We developed a query to target such interactions and the metabolites produced by the protective allelopathic plants.

**Figure 7:**
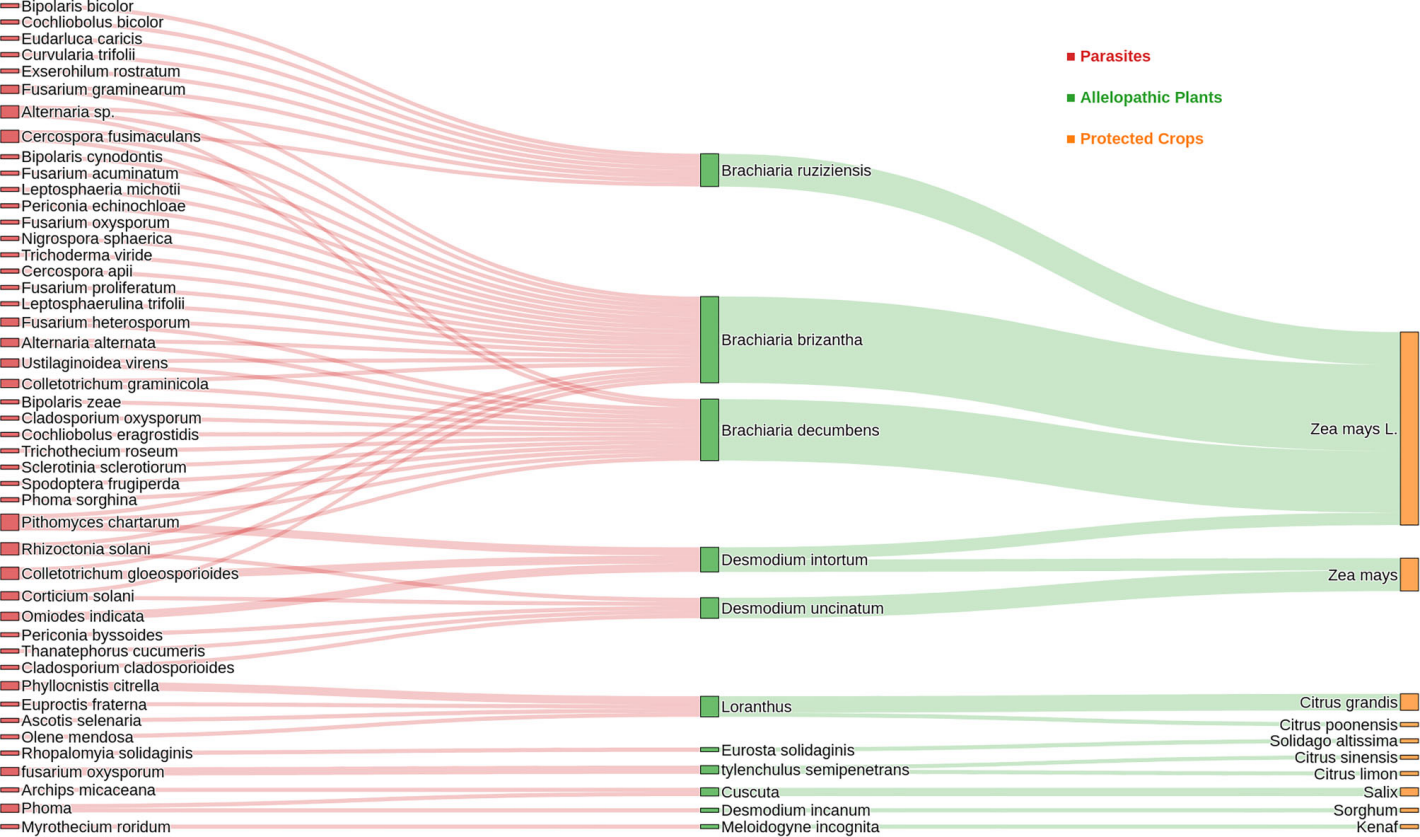
CS4–Sankey plot representing the flow of allelopathy interactions in push–pull agriculture. Parasites are shown in red, allelopathic plants in green, and protected crops in orange.

In the original GloBI dataset, several fungal genera (e.g., *Fusarium, Puccinia, Claviceps, Sclerotinia sclerotiorum*) were misidentified as allelopath, likely resulting from automated inverse relationship generation, where Plant “hasPathogen” Fungus was inverted to Fungus “allelopathOf” Plant. In the result obtained for CS4, we removed 55 interactions (45% of the original dataset) where fungi were incorrectly classified as allelopaths, as these organisms represent direct pathogenic relationships rather than a plant-mediated chemical interference. After applying this filter, the results show 67 tripartite interactions involving 46 parasites, 11 allelopathic plants, and 10 crops (Fig. 7, Supplementary Table S8).

#### CS5: Retrieve traits and metabolites for organisms for which traits from the Leaf Economic Spectrum (LES) are available

Subject—Theoretical ecology

Queries are described in [153] and [155] (Table 2).

The LES is a foundational concept in plant ecology, describing coordinated variation in leaf traits across species, typically forming a trade-off between resource-acquisitive and resource-conservative strategies [81, 204]. Resource-acquisitive species have traits like high specific leaf area (SLA), high nitrogen content, and short leaf lifespan, which are optimized for rapid growth in resource-rich environments. Resource-conservative species show traits like low SLA, low nutrient content, and long leaf lifespan, which are better suited for resource-poor environments with conservative growth. These relationships are global, cross-taxonomic, and continuous. They help predict ecosystem functions, species distributions, and responses to environmental changes.

We developed a query to retrieve values for 4 traits (including their subcategories totaling to 6) relevant for LES—specific leaf area, leaf dry matter content, leaf nitrogen content, and leaf phosphorus content. Further, we developed another query to retrieve metabolites produced by species with data for 4 LES traits available in the knowledge graph. While we were unable to identify plant species with both metabolite data and complete trait coverage across all 4 categories, this limitation itself reinforces the value of our knowledge graph approach and the importance of continued data integration efforts.

Table 4 depicts LES trait data from 1,058 plant species with metabolite profiles from 627 species, identifying 49 species (with incomplete trait coverage) with both datasets available for comparative analysis. The data encompassed 6 key functional traits across 2,324 measurements and 11,313 unique metabolites across 16,587 associations.

**Table 4:** Metrics for CS4

Metric	Value
Unique species with traits	1,058
Unique traits	6
Unique species with metabolites	627
Unique metabolites	11,313
Unique species with both traits and metabolites	49
Total trait records	2,324
Total metabolite records	16,587

At the time of writing this manuscript, the results of our queries reflect the state of METRIN-KG and the federated sources available. As these underlying datasets are continuously updated and expanded, the query outputs presented here may evolve over time. All scripts used to generate the tables and figures for case studies are available in the METRIN-KG GitHub repository [128].

## Conclusions

We provide METRIN-KG, aiming to integrate seemingly disconnected databases of plant metabolomes, plant traits, and interactions. To facilitate exploration of this resource, we provide a way to search for those connections through a user interface. We aspire to draw the attention of researchers in areas of drug discovery, ecology, human health, biodiversity conservation, and agriculture to the power of knowledge graphs in integrating life sciences datasets. We wish to increase awareness among researchers to include as varied data as possible, so as to approach research questions from multiple perspectives. In this context, we provide representative case studies to retrieve data for different research projects. We plan to extend METRIN-KG to incorporate the full breadth of metabolome data from plants in the near future. In the long term, we plan to extend it, adding more information on metabolite data for other kingdoms beyond Plantae, while updating the trait and interaction data from TRY and GloBI, respectively, as new versions are released. We hope that our efforts will encourage other researchers to use and contribute to this resource.

## Availability of source code and requirements

**METRIN-KG**

Project name: metrin-kg

Project homepage: https://GitHub.com/earth-metabolome-initiative/metrin-kg

License: GPL-3.0 license

Operating system(s): Ubuntu 24.04.2 LTS

Programming language: Python

Hardware requirements: 32 GB RAM, 500 GB disk capacity

RRID:SCR_027914

biotools: metrin_kg

METRIN-KG’s code is also available through Zenodo [205].

**Earth Metabolome Initiative Ontology**

Project name: Earth Metabolome Initiative Ontology, illustrated with an example of knowledge graph construction

Project homepage: https://GitHub.com/earth-metabolome-initiative/earth_metabolome_ontology

License: Software code under GPL-3.0 license, Ontology under CC0-1.0 license

Operating system(s): modern Linux/Windows/Unix distribution (64–bit OS)

Programming language: OWL, Python

Hardware requirements: any x86_64 or ARM64 CPU with at least 2 cores and a minimum of 8 GB RAM and 60 GB disk are recommended for functional setups

RRID:SCR_027915

biotools: earth_metabolome_initiative_ontology

## Additional files

**Supplementary Table S1**. List of taxonomy databases used in GloBI.

**Supplementary Table S2**. List of ontologies used in METRIN-KG.

**Supplementary Table S3**. Column descriptions of data retrieved from the TRY database. Column names and descriptions were retained from the files provided by TRY database managers

**Supplementary Table S4**. Metrics for the TRY database and GloBI.

**Supplementary Table S5**. Column descriptions of data retrieved from GloBI. Column names and descriptions obtained from the GloBI dataset template repository [138].

**Supplementary Table S6**. CS2—summary of species distributions.

**Supplementary Table S7**. CS2—summary of trait distributions.

**Supplementary Table S8**. Filtered data without fungi species for CS4.

**Supplementary Data.pdf**.

## Abbreviations

AO: Agronomy Ontology; APO: Ascomycete Phenotype Ontology; BRENDA: Braunschweig Enzyme Database; BSPO: Biological Spatial Ontology; BTO: BRENDA Tissue Ontology; CEPH: Cephalopod Ontology; ChEBI: Chemical Entities of Biological Interest; CL: Cell Ontology; CLAO: Collembola Anatomy Ontology; CS: case study; CSV: comma-separated values; DBGI: Digital Botanical Gardens Initiative; DDANAT: Dictyostelium Discoideum Anatomy; ECOCORE: Ecology Core Ontology; EFO: Experimental Factor Ontology; EMI: Earth Metabolome Initiative; ENPKG: Experimental Natural Products Knowledge Graph; ENVO: Environment Ontology; FAIR: Findable, Accessible, Interoperable, and Reusable; FAO: Fungal Anatomy Ontology; FLOPO: Flora Phenotype Ontology; FMA: Foundational Model of Anatomy; FOODON: Food Ontology; GallOnt: ontology for plant gall phenotypes; GloBI: Global Biotic Interactions; GO: Gene Ontology; HAO: Hymenoptera Anatomy Ontology; IDO: Infectious Disease Ontology; IDOMAL: Infectious Disease Ontology for Malaria; IRI: Internationalized Resource Identifier; IUCN: International Union for Conservation of Nature; KG: knowledge graph; LES: Leaf Economic Spectrum; LMA: leaf dry mass per area; METRIN-KG: MEtabolomes, TRaits, and INteractions-Knowledge Graph; NCIT: National Cancer Institute Thesaurus; OBI: Ontology for Biomedical Investigations; OGC: Open Geospatial Consortium; OMIT: Ontology for MIcroRNA Target Prediction; OWL: Web Ontology Language; PATO: Phenotype and Trait Ontology; PHIPO: Pathogen–Host Interaction Phenotype Ontology; PNUE: Photosynthetic Nitrogen Use Efficiency; PO: Plant Ontology; PORO: Porifera Ontology; PURL: Persistent Uniform Resource Locators; QUDT: Quantities, Units, Dimensions, and Types; RDF: Resource Description Framework; RO: Relation Ontology; SHACL: Shapes Constraint Language; SKOS: Simple Knowledge Organization System; SLA: specific leaf area; SOSA: Sensor, Observation, Sample, and Actuator; SPARQL: SPARQL Protocol and RDF Query Language; SSD: Stem Specific Density; SQL: Structured Query Language; TGMA: mosquito gross anatomy ontology; TPU: Triose Phosphate Utilisation; TSV: tab-separated values; UBERON: Uber-Anatomy Ontology; UI: user interface; URL: Uniform Resource Locators; VoID: Vocabulary of Interlinked Datasets; W3C: World Wide Web Consortium; WGS84: World Geodetic System 1984; WUE: Water Use Efficiency.

## Supplementary Material

giag051_Supplemental_Files

giag051_Authors_Response_To_Reviewer_Comments_original_submission

giag051_GIGA-D-25-00342_original_submission

giag051_GIGA-D-25-00342_Revision_1

giag051_Reviewer_1_Report_original_submission Reviewer 1 -- 9/29/2025

giag051_Reviewer_2_Report_original_submission Reviewer 2 -- 11/19/2025

giag051_Reviewer_2_Report_revision_1 Reviewer 2 -- 3/27/2026

## Data Availability

All input and output files used in mapping taxonomy, metadata, ontology, and knowledge graph construction as well as RDF files are available on EMI’s Zenodo repository for METRIN-KG (CC0-1.0) [104] and TRY (CC-BY-4.0) [95].

## References

[1] Hortal J, de Bello B, Diniz-Filho JAF et al. Seven shortfalls that beset large-scale knowledge of biodiversity. Annu Rev Ecol Evol Syst. 2015;46:523–49. 10.1146/annurev-ecolsys-112414-054400.

[2] Cardoso P, Erwin T, Borges P et al. The seven impediments in invertebrate conservation and how to overcome them. Biol Conserv. 2011;144:2647–55. 10.1016/j.biocon.2011.07.024.

[3] Pollock L J, Kitzes J, Beery S et al. Harnessing artificial intelligence to fill global shortfalls in biodiversity knowledge. Nat Rev Biodivers. 2025;1:166–82. 10.1038/s44358-025-00022-3.

[4] Walker TWN, Alexander J M, Allard P-M et al. Functional traits 2.0: the power of the metabolome for ecology. J Ecol. 2022;110:4–20. 10.1111/1365-2745.13826.

[5] Hilker M . New synthesis: parallels between biodiversity and chemodiversity. J Chem Ecol. 2014;40:225–26. 10.1007/s10886-014-0402-8. 24590508

[6] Schuman M C, Baldwin I T. The layers of plant responses to insect herbivores. Annu Rev Entomol. 2016;61:373–94. 10.1146/annurev-ento-010715-023851. 26651543

[7] Wink M . Evolution of secondary metabolites from an ecological and molecular phylogenetic perspective. Phytochemistry. 2003;64:3–19. 10.1016/S0031-9422(03)00300-5. 12946402

[8] Hart C E, Gadiya Y, Kind T et al. Defining the limits of plant chemical space: challenges and estimations. Gigascience. 2025;14:giaf033. 10.1093/gigascience/giaf033. PMC11970369 40184432

[9] Van Dam N M, Van Der Meijden E. A role for metabolomics in plant ecology. In: Hall R D, editor. Annual Plant Reviews. Vol. 43. Wiley; 2011. 10.1002/9781444339956.ch4.

[10] Weckwerth W . Metabolomics in systems biology. Annu Rev Plant Biol. 2003;54:669–89. 10.1146/annurev.arplant.54.031902.135014. 14503007

[11] Echeverri A, Karp D S, Naidoo R et al. Can avian functional traits predict cultural ecosystem services?. People Nature. 2020;2:138–51. 10.1002/pan3.10058.

[12] Wong MKL, Guénard B, Lewis O T. Trait-based ecology of terrestrial arthropods. Biol Rev. 2019;94:999–1022. 10.1111/brv.12488. 30548743 PMC6849530

[13] Lundgren E J, Schowanek S D, Rowan J et al. Functional Traits of the World’s Late Quaternary Large-Bodied Avian and Mammalian Herbivores. Sci Data. 2021;8:17. 10.1038/s41597-020-00788-5. 33473149 PMC7817692

[14] Kunin W E, Vergeer P, Kenta T et al. Variation at range margins across multiple spatial scales: environmental temperature, population genetics and metabolomic phenotype. Proc R Soc B. 2009;276:1495–506. 10.1098/rspb.2008.1767. PMC2677219 19324821

[15] Dwyer J M, Hobbs R J, Mayfield M M. Specific leaf area responses to environmental gradients through space and time. Ecology. 2014;95:399–410. 10.1890/13-0412.1. 24669733

[16] Adler P B, Salguero-Gómez R, Compagnoni A et al. Functional traits explain variation in plant life history strategies. Proc Natl Acad Sci USA. 2014;111:740–45. 10.1073/pnas.1315179111. 24379395 PMC3896207

[17] Pistón N, de Bello F, Dias ATC et al. Multidimensional ecological analyses demonstrate how interactions between functional traits shape fitness and life history strategies. J Ecol. 2019;107:2317–28. 10.1111/1365-2745.13190.

[18] Walker TWN, Weckwerth W, Bragazza L et al. Plastic and genetic responses of a common sedge to warming have contrasting effects on carbon cycle processes. Ecol Lett. 2019;22:159–69. 10.1111/ele.13178. 30556313 PMC6334510

[19] van der Plas F, Schröder-Georgi T, Weigelt A et al. Plant traits alone are poor predictors of ecosystem properties and long-term ecosystem functioning. Nat Ecol Evol. 2020;4:1602–11. 10.1038/s41559-020-01316-9. 33020598

[20] Firn J, McGree J M, Harvey E et al. Leaf nutrients, not specific leaf area, are consistent indicators of elevated nutrient inputs. Nat Ecol Evol. 2019;3:400–6. 10.1038/s41559-018-0790-1. 30718853

[21] Laughlin D C, Gremer J R, Adler P B et al. The net effect of functional traits on fitness. Trends Ecol Evol. 2020;35:1037–47. 10.1016/j.tree.2020.07.010. 32807503

[22] Eisenhauer N, Bonfante P, Buscot F et al. Biotic interactions as mediators of context-dependent biodiversity-ecosystem functioning relationships. Res Ideas Outcomes. 2022;8:e85873. 10.3897/rio.8.e85873.

[23] Maestre F T, Bowker M A, Escolar C et al. Do biotic interactions modulate ecosystem functioning along stress gradients? Insights from semi-arid plant and biological soil crust communities. Phil Trans R Soc B. 2010;365:2057–70. 10.1098/rstb.2010.0016. 20513714 PMC2880128

[24] Jayaramaiah R H, Egidi E, Macdonald C A et al. Linking biodiversity and biotic interactions to ecosystem functioning. J Sust Agri Env. 2024;3:e12119. 10.1002/sae2.12119.

[25] de Vries S, Feussner I. Biotic interactions, evolutionary forces and the pan-plant specialized metabolism. Philos Trans R Soc B Biol Sci. 2024;379:20230362. 10.1098/rstb.2023.0362. PMC11449213 39343027

[26] Defossez E, Pitteloud C, Descombes P et al. Spatial and evolutionary predictability of phytochemical diversity. Proc Natl Acad Sci USA. 2021;118:e2013344118. 10.1073/pnas.2013344118. 33431671 PMC7826413

[27] Agrawal A A, Fishbein M, Halitschke R et al. Evidence for adaptive radiation from a phylogenetic study of plant defenses. Proc Natl Acad Sci USA. 2009;106:18067–72. 10.1073/pnas.0904862106. 19805160 PMC2775318

[28] Ehrlich P R, Raven P H. Butterflies and plants: a study in coevolution. Evolution. 1964;18:586. 10.2307/2406212.

[29] Bennett R N, Wallsgrove R M. Secondary metabolites in plant defence mechanisms. New Phytol. 1994;127:617–33. 10.1111/j.1469-8137.1994.tb02968.x. 33874382

[30] Walker TWN, Schrodt F, Allard P-M et al. Leaf metabolic traits reveal hidden dimensions of plant form and function. Sci Adv. 2023;9:eadi4029. 10.1126/sciadv.adi4029. 37647404 PMC10468135

[31] Deng P, Yin R, Wang H et al. Comparative analyses of functional traits based on metabolome and economic traits variation of Bletilla striata: contribution of intercropping. Front Plant Sci. 2023;14:1147076. 10.3389/fpls.2023.1147076. 37008465 PMC10064063

[32] Wei J, Wang A, Li R et al. Metabolome-wide association studies for agronomic traits of rice. Heredity. 2018;120:342–55. 10.1038/s41437-017-0032-3. 29225351 PMC5842221

[33] Delory B M, Callaway R M, Semchenko M. A trait-based framework linking the soil metabolome to plant–soil feedbacks. New Phytol. 2024;241:1910–21. 10.1111/nph.19490. 38124274

[34] Shi T, Zhu A, Jia J et al. Metabolomics analysis and metabolite-agronomic trait associations using kernels of wheat (Triticum aestivum) recombinant inbred lines. Plant J. 2020;103:279–92. 10.1111/tpj.14727. 32073701 PMC7383920

[35] Green S J, Brookson C B, Hardy N A et al. Trait-based approaches to global change ecology: moving from description to prediction. Proc R Soc B. 2022;289:20220071. 10.1098/rspb.2022.0071. PMC8924753 35291837

[36] Walter H E, Pagel J, Cooksley H et al. Effects of biotic interactions on plant fecundity depend on spatial and functional structure of communities and time since disturbance. J Ecol. 2023;111:110–24. 10.1111/1365-2745.14018.

[37] Pueyo Y, Kéfi S, Díaz-Sierra R et al. The role of reproductive plant traits and biotic interactions in the dynamics of semi-arid plant communities. Theor Popul Biol. 2010;78:289–97. 10.1016/j.tpb.2010.09.001. 20875441

[38] Gaüzère P, O’Connor L, Botella C et al. The diversity of biotic interactions complements functional and phylogenetic facets of biodiversity. Curr Biol. 2022;32:2093–100.e3. 10.1016/j.cub.2022.03.009. 35334226

[39] Tenenboim H, Brotman Y. Omic relief for the biotically stressed: metabolomics of plant biotic interactions. Trends Plant Sci. 2016;21:781–91. 10.1016/j.tplants.2016.04.009. 27185334

[40] Gupta S, Schillaci M, Roessner U. Metabolomics as an emerging tool to study plant–microbe interactions. Emerg Top Life Sci. 2022;6:175–83. 10.1042/ETLS20210262. 35191478 PMC9023012

[41] Henderson D, Sedio B E, Tello J S et al. Ecological metabolomics of tropical tree communities across an elevational gradient: implications for chemically-mediated biotic interactions and species diversity. Biorxiv. 2023. 10.1101/2023.10.04.560880. Accessed 30 August 2024.

[42] Maag D, Erb M, Glauser G. Metabolomics in plant–herbivore interactions: challenges and applications. Entomologia Exp Applicata. 2015;157:18–29. 10.1111/eea.12336.

[43] Majumdar S, Kaur H, Rinella M J et al. Synergistic effects of canopy chemistry and autogenic soil biota on a global invader. J Ecol. 2023;111:1497–513. 10.1111/1365-2745.14113.

[44] Semchenko M, Nettan S, Sepp A et al. Soil biota and chemical interactions promote co-existence in co-evolved grassland communities. J Ecol. 2019;107:2611–22. 10.1111/1365-2745.13220.

[45] Burghardt K T, Bradford M A, Schmitz O J. Acceleration or deceleration of litter decomposition by herbivory depends on nutrient availability through intraspecific differences in induced plant resistance traits. J Ecol. 2018;106:2380–94. 10.1111/1365-2745.13002.

[46] Heinen R, Biere A, Bezemer T M. Plant traits shape soil legacy effects on individual plant–insect interactions. Oikos. 2020;129:261–73. 10.1111/oik.06812.

[47] De Long J R, Heinen R, Hannula S E et al. Plant-litter-soil feedbacks in common grass species are slightly negative and only marginally modified by litter exposed to insect herbivory. Plant Soil. 2023;485:227–44. 10.1007/s11104-022-05590-3.

[48] Eissenstat D M, Kucharski J M, Zadworny M et al. Linking root traits to nutrient foraging in arbuscular mycorrhizal trees in a temperate forest. New Phytol. 2015;208:114–24. 10.1111/nph.13451. 25970701

[49] Stiblíková P, Klimeš A, Cahill J F et al. Interspecific differences in root foraging precision cannot be directly inferred from species’ mycorrhizal status or fine root economics. Oikos. 2023;2023:e08995. 10.1111/oik.08995.

[50] Xia M, Valverde-Barrantes O J, Suseela V et al. Coordination between compound-specific chemistry and morphology in plant roots aligns with ancestral mycorrhizal association in woody angiosperms. New Phytol. 2021;232:1259–71. 10.1111/nph.17561. 34137048

[51] Kong C-H, Zhang S-Z, Li Y-H et al. Plant neighbor detection and allelochemical response are driven by root-secreted signaling chemicals. Nat Commun. 2018;9:3867. 10.1038/s41467-018-06429-1. 30250243 PMC6155373

[52] Li L, Li S-M, Sun J-H et al. Diversity enhances agricultural productivity via rhizosphere phosphorus facilitation on phosphorus-deficient soils. Proc Natl Acad Sci USA. 2007;104:11192–96. 10.1073/pnas.0704591104. 17592130 PMC1899187

[53] Steinauer K, Thakur M P, Emilia Hannula S et al. Root exudates and rhizosphere microbiomes jointly determine temporal shifts in plant-soil feedbacks. Plant Cell Environ. 2023;46:1885–99. 10.1111/pce.14570. 36794528

[54] Henderson D, Tello J S, Cayola L et al. Testing the role of biotic interactions in shaping elevational diversity gradients: an ecological metabolomics approach. Ecology. 2025;106:e70069. 10.1002/ecy.70069. 40207495

[55] Bovay B, Descombes P, Chittaro Y et al. Adapting to change: exploring the consequences of climate-induced host plant shifts in two specialist lepidoptera species. Ecol Evol. 2024;14:e11596. 10.1002/ece3.11596. 38932969 PMC11199125

[56] Sierra A M, Meléndez O, Bethancourt R et al. Leaf endophytes relationship with host metabolome expression in tropical gymnosperms. J Chem Ecol. 2024;50:815–29. 10.1007/s10886-024-01511-z. 38809282

[57] Mleziva A D, Ngumbi E N. Comparative analysis of defensive secondary metabolites in wild teosinte and cultivated maize under flooding and herbivory stress. Physiol Plant. 2024;176:e14216. 10.1111/ppl.14216. 38366721

[58] Gallon M E, Muchoney N D, Smilanich A M. Viral infection induces changes to the metabolome, immune response and development of a generalist insect herbivore. J Chem Ecol. 2024;50:152–67. 10.1007/s10886-024-01472-3. 38353894

[59] Contreras-Cornejo H A, Schmoll M, Esquivel-Ayala B A et al. Mechanisms for plant growth promotion activated by Trichoderma in natural and managed terrestrial ecosystems. Microbiol Res. 2024;281:127621. 10.1016/j.micres.2024.127621. 38295679

[60] Yasmin F, Cowie A E, Zerbe P. Understanding the chemical language mediating maize immunity and environmental adaptation. New Phytol. 2024;243:2093–101. 10.1111/nph.20000. 39049575

[61] Ehlers B K, Berg M P, Staudt M et al. Plant secondary compounds in soil and their role in belowground species interactions. Trends Ecol Evol. 2020;35:716–30. 10.1016/j.tree.2020.04.001. 32414604

[62] Delory B M, Delaplace P, Fauconnier M-L et al. Root-emitted volatile organic compounds: can they mediate belowground plant-plant interactions?. Plant Soil. 2016;402:1–26. 10.1007/s11104-016-2823-3.

[63] Semchenko M, Barry K E, de Vries F T et al. Deciphering the role of specialist and generalist plant–microbial interactions as drivers of plant–soil feedback. New Phytol. 2022;234:1929–44. 10.1111/nph.18118. 35338649

[64] Ninkovic V, Markovic D, Rensing M. Plant volatiles as cues and signals in plant communication. Plant Cell Environ. 2021;44:1030–43. 10.1111/pce.13910. 33047347 PMC8048923

[65] Moore B D, Andrew R L, Külheim C et al. Explaining intraspecific diversity in plant secondary metabolites in an ecological context. New Phytol. 2014;201:733–50. 10.1111/nph.12526. 24117919

[66] Bilas R D, Bretman A, Bennett T. Friends, neighbours and enemies: an overview of the communal and social biology of plants. Plant Cell Environ. 2021;44:997–1013. 10.1111/pce.13965. 33270936

[67] Wang N-Q, Kong C-H, Wang P et al. Root exudate signals in plant–plant interactions. Plant Cell Environ. 2021;44:1044–58. 10.1111/pce.13892. 32931018

[68] Rutz A, Sorokina M, Galgonek J et al. The LOTUS initiative for open knowledge management in natural products research. eLife. 2022;11:e70780. 10.7554/eLife.70780. 35616633 PMC9135406

[69] Gaudry A, Pagni M, Mehl F et al. A sample-centric and knowledge-driven computational framework for natural products drug discovery. ACS Cent Sci. 2024;10:494–510. 10.1021/acscentsci.3c00800. 38559298 PMC10979503

[70] Kim S, Chen J, Cheng T et al. PubChem 2025 update. Nucleic Acids Res. 2025;53:D1516–25. 10.1093/nar/gkae1059. 39558165 PMC11701573

[71] Zdrazil B, Felix E, Hunter F et al. The ChEMBL Database in 2023: a drug discovery platform spanning multiple bioactivity data types and time periods. Nucleic Acids Res. 2024;52:D1180–92. 10.1093/nar/gkad1004. 37933841 PMC10767899

[72] Afendi F M, Okada T, Yamazaki M et al. KNApSAcK family databases: integrated metabolite–plant species databases for multifaceted plant research. Plant Cell Physiol. 2012;53:e1. 10.1093/pcp/pcr165. 22123792

[73] The Earth Metabolome Initiative. https://www.earthmetabolome.org/. (2023) Accessed 14 February 2026.

[74] The Digital Botanical Gardens Initiative . https://digital-botanical-gardens-initiative.github.io/dbgi-green-paper/. (2022) Accessed 11 June 2025.

[75] Wilkinson M D, Dumontier M, Aalbersberg I J et al. The FAIR Guiding Principles for scientific data management and stewardship. Sci Data. 2016;3:160018. 10.1038/sdata.2016.18. 26978244 PMC4792175

[76] Poelen J H, Simons J D, Mungall C J. Global biotic interactions: an open infrastructure to share and analyze species-interaction datasets. Ecol Inform. 2014;24:148–59. 10.1016/j.ecoinf.2014.08.005.

[77] BiotXplorer . https://biotxplorer.sibils.org/. Accessed 4 June 2025.

[78] Page R . Towards a biodiversity knowledge graph. Res Ideas Outcomes. 2016;2:e8767. 10.3897/rio.2.e8767.

[79] Kattge J, Díaz S, Lavorel S et al. TRY—a global database of plant traits. Global Change Biol. 2011;17:2905–35. 10.1111/j.1365-2486.2011.02451.x.

[80] Kattge J, Bönisch G, Díaz S et al. TRY plant trait database—enhanced coverage and open access. Global Change Biol. 2020;26:119–88. 10.1111/gcb.14904. 31891233

[81] Wright I J, Reich P B, Westoby M et al. The worldwide leaf economics spectrum. Nature. 2004;428:821–27. 10.1038/nature02403. 15103368

[82] Nordt B, Hensen I, Bucher S F et al. The PhenObs initiative: a standardised protocol for monitoring phenological responses to climate change using herbaceous plant species in botanical gardens. Funct Ecol. 2021;35:821–34. 10.1111/1365-2435.13747.

[83] Caspi R, Billington R, Keseler I M et al. The MetaCyc database of metabolic pathways and enzymes—a 2019 update. Nucleic Acids Res. 2020;48:D445–53. 10.1093/nar/gkz862. 31586394 PMC6943030

[84] Karp P D, Paley S, Caspi R et al. The EcoCyc Database (2023). EcoSal Plus. 2023;11:eesp–0002-2023. 10.1128/ecosalplus.esp-0002-2023. PMC10729931 37220074

[85] Karp P D, Billington R, Caspi R et al. The BioCyc collection of microbial genomes and metabolic pathways. Brief Bioinform. 2019;20:1085–93. 10.1093/bib/bbx085. 29447345 PMC6781571

[86] Singh K, Maurya H, Singh P et al. DISPEL: database for ascertaining the best medicinal plants to cure human diseases. Database. 2023;2023:baad073. 10.1093/database/baad073. 37847815 PMC10581335

[87] Youn J, Li F, Simmons G et al. Automated knowledge extraction of food and chemicals from literature. Biorxiv. 2024. 10.1101/2024.05.16.594596. Accessed 2 Sep 2024. 39216404

[88] The Earth Metabolome Initiative (EMI) ontology. https://w3id.org/emi. Accessed 23 August 2025.

[89] The Earth Metabolome Initiative (EMI) ontology. https://www.earthmetabolome.org/earth_metabolome_ontology/. Accessed 23 August 2025.

[90] Calvanese D, Lanti D, Mendes De Farias T et al. Accessing scientific data through knowledge graphs with Ontop. Patterns. 2021;2:100346. 10.1016/j.patter.2021.100346. 34693372 PMC8515008

[91] Calvanese D, Cogrel B, Komla-Ebri S et al. Ontop: answering SPARQL queries over relational databases. Semantic Web. 2016;8:471–87. 10.3233/SW-160217.

[92] Xiao G, Lanti D, Kontchakov R et al. The Virtual Knowledge Graph System Ontop. In: Pan J Z, et al. editor. The Semantic Web – ISWC 2020. ISWC 2020. Lecture Notes in Computer Science. Vol. 12507. 259–77. Cham: Springer. 10.1007/978-3-030-62466-8_17.

[93] RDFlib . https://rdflib.dev/. Accessed 6 June 2025.

[94] TRY Data Explorer . https://www.try-db.org/TryWeb/dp2.php. Accessed 23 August 2025.

[95] Tandon D . Plant traits data from TRY database (raw data for METRIN-KG) (Version v1). Zenodo. 10.5281/zenodo.17079465. Accessed 15 February 2026.

[96] Community GloBI . Global biotic interactions: interpreted data products (Version 0.8). Zenodo. 10.5281/zenodo.14640564. Accessed 4 June 2025.

[97] GloBI data . https://www.globalbioticinteractions.org/data. Accessed 4 June 2025.

[98] GloBI GitHub repository . https://GitHub.com/globalbioticinteractions. Accessed 4 June 2025.

[99] GloBI datasets. https://www.globalbioticinteractions.org/datasets. Accessed 4 June 2025.

[100] Bast H, Buchhold B. QLever: a query engine for efficient SPARQL+text search. In: Proc 2017 ACM Conf Inf Knowl Manag. New York, NY: Association for Computing Machinery. 2017, 647–56. 10.1145/3132847.3132921.

[101] Qlever Wikidata SPARQL endpoint . https://qlever.dev/wikidata. Accessed 14 February 2026.

[102] Query to map wikidata identifiers to other taxonomies. https://qlever.dev/wikidata/S2gD0b. Accessed 14 February 2026.

[103] Query to retrieve lineage from Wikidata identifiers. https://qlever.dev/wikidata/66ksWA. Accessed 14 February 2026.

[104] Tandon D, Mendes de Farias T, Allard P-M et al. METRIN-KG Data (Version v7). Zenodo. 10.5281/zenodo.19485732. Accessed 18 February 2026.

[105] Uber-Anatomy Ontology . https://purl.obolibrary.org/obo/uberon.owl. Accessed 28 May 2025.

[106] Plant Ontology . https://purl.obolibrary.org/obo/po.owl. Accessed 28 May 2025.

[107] Environment Ontology . https://purl.obolibrary.org/obo/envo.owl. Accessed 28 May 2025.

[108] Gene Ontology . https://purl.obolibrary.org/obo/go.owl. Accessed 28 May 2025.

[109] Phenotype and Trait Ontology . https://purl.obolibrary.org/obo/pato.owl. Accessed 28 May 2025.

[110] METRIN-KG ontology matching at main · earth-metabolome-initiative/metrin-kg. GitHub. https://GitHub.com/earth-metabolome-initiative/metrin-kg/tree/main/src/ontology_matching. Accessed 14 February 2026.

[111] Lamy J-B . Owlready: ontology-oriented programming in Python with automatic classification and high level constructs for biomedical ontologies. Artif Intell Med. 2017;80:11–28. 10.1016/j.artmed.2017.07.002. 28818520

[112] Reimers N, Gurevych I. Sentence-BERT: sentence embeddings using siamese BERT-networks. arXiv. 2019. 10.48550/arXiv.1908.10084. Accessed 23 December 2024.

[113] QUDT units vocabulary. https://qudt.org/doc/2025/01/DOC_VOCAB-UNITS-ALL.html. Accessed 23 August 2025.

[114] FAIRsharing Team . FAIRsharing record for: Quantities, Units, Dimensions and Types. FAIRsharing. 2015. 10.25504/FAIRSHARING.D3PQW7. Accessed 4 June 2025.

[115] W3C. https://www.w3.org/. Accessed 23 August 2025.

[116] Janowicz K, Haller A, Cox SJD et al. SOSA: a lightweight ontology for sensors, observations, samples, and actuators. J Web Semant. 2019;56:1–10. 10.1016/j.websem.2018.06.003.

[117] Advancing Geospatial Standards and Technology | OGC. Open Geospatial Consort. https://www.ogc.org/. Accessed 4 June 2025.

[118] OBO Foundry . https://obofoundry.org/ontology/ro.html. Accessed 23 August 2025.

[119] SKOS Simple Knowledge Organization System Namespace Document—HTML Variant, 18 August 2009 Recommendation Edition. https://www.w3.org/2009/08/skos-reference/skos.html. Accessed 4 June 2025.

[120] W3C Semantic Web Interest Group: Basic Geo (WGS84 lat/long) Vocabulary. https://www.w3.org/2003/01/geo/. Accessed 4 June 2025.

[121] The ENPKG RDF vocabulary. https://enpkg.commons-lab.org/doc/index.html. Accessed 23 August 2025.

[122] Earth Metabolome Ontology GitHub repository. https://GitHub.com/earth-metabolome-initiative/earth_metabolome_ontology. Accessed 15 February 2026.

[123] Allard P-M, Gaudry A. Input and enriched files for the pf1600 dataset—ENPKG (Version 1.0). Zenodo. 10.5281/zenodo.10827917. Accessed 4 June 2025.

[124] Allard P-M, Gaudry A, Quirós-Guerrero L-M et al. Open and reusable annotated mass spectrometry dataset of a chemodiverse collection of 1,600 plant extracts. Gigascience. 2023;12:giac124. 10.1093/gigascience/giac124. PMC9845059 36649739

[125] MySQL :: MySQL 8.2 Release Notes. https://dev.mysql.com/doc/relnotes/mysql/8.2/en/. Accessed 6 June 2025.

[126] Earth Metabolome Initiative Ontology Ontop Mapping. The Earth Metabolome Initiative. https://GitHub.com/earth-metabolome-initiative/earth_metabolome_ontology/blob/main/ontop_config/emi-v1/emi-v1.obda. Accessed 23 August 2025.

[127] Krech D, Grimnes G A, Higgins G et al. RDFLib (Version 7.0.0). Zenodo. 10.5281/zenodo.8206632. Accessed 3 March 2024.

[128] METRIN-KG GitHub repository. The Earth Metabolome Initiative. https://github.com/earth-metabolome-initiative/metrin-kg. Accessed 15 February 2026.

[129] Qlever-control GitHub repository. University of Freiburg: Algorithms and Data Structures Group. https://github.com/qlever-dev/qlever-control. Accessed 15 February 2026.

[130] METRIN-KG SPARQL endpoint. https://kg.earthmetabolome.org/metrin/. Accessed 15 February 2026.

[131] METRIN-KG Qlever SPARQL endpoint API. https://kg.earthmetabolome.org/metrin/api. Accessed 15 February 2026.

[132] Programmatic access to METRIN-KG SPARQL endpoint. GitHub. https://github.com/earth-metabolome-initiative/metrin-kg/wiki/How-to-programatically-access-METRIN%E2%80%90KG%27s-SPARQL-endpoint%3F. Accessed 15 February 2026.

[133] METRIN-KG SPARQL underlying endpoint. https://qlever.earthmetabolome.org/metrin-kg/. Accessed 15 February 2026.

[134] Bast H, Kalmbach J, Klumpp T et al. Efficient SPARQL autocompletion via SPARQL. arXiv. 2021. 10.48550/arXiv.2104.14595. Accessed 4 June 2025.

[135] Qlever-ui GitHub repository. University of Freiburg: Algorithms and Data Structures Group. https://github.com/qlever-dev/qlever-ui. Accessed 15 February 2026.

[136] Fork of Qlever-ui GitHub repository. The Earth Metabolome Initiative. https://github.com/earth-metabolome-initiative/qlever-ui. Accessed 15 February 2026.

[137] METRIN-KG metrics-1 query. https://kg.earthmetabolome.org/metrin/metrics_1. Accessed 15 February 2026.

[138] GloBI dataset template data format. https://github.com/globalbioticinteractions/template-dataset#data-format-and-dictionary. Accessed 4 June 2025.

[139] METRIN-KG metrics-2 query. https://kg.earthmetabolome.org/metrin/metrics_2. Accessed 15 February 2026.

[140] METRIN-KG metrics-3 query. https://kg.earthmetabolome.org/metrin/metrics_3. Accessed 15 February 2026.

[141] METRIN-KG example query-11. https://kg.earthmetabolome.org/metrin/11. Accessed 15 February 2026.

[142] METRIN-KG example query-11 versioned. GitHub. https://kg.earthmetabolome.org/metrin/11/v/53581b1. Accessed 15 February 2026.

[143] METRIN-KG example query-12. https://kg.earthmetabolome.org/metrin/12. Accessed 15 February 2026.

[144] METRIN-KG example query-12 versioned. GitHub. https://kg.earthmetabolome.org/metrin/12/v/53581b1. Accessed 15 February 2026.

[145] METRIN-KG example query-13. https://kg.earthmetabolome.org/metrin/13. Accessed 15 February 2026.

[146] METRIN-KG example query-13 versioned. GitHub. https://kg.earthmetabolome.org/metrin/13/v/53581b1. Accessed 15 February 2026.

[147] METRIN-KG example query-14. https://kg.earthmetabolome.org/metrin/14. Accessed 15 February 2026.

[148] METRIN-KG example query-14 versioned. GitHub. https://kg.earthmetabolome.org/metrin/14/v/53581b1. Accessed 15 February 2026.

[149] METRIN-KG example query-20. https://kg.earthmetabolome.org/metrin/20. Accessed 15 February 2026.

[150] METRIN-KG example query-20 versioned. GitHub. https://kg.earthmetabolome.org/metrin/20/v/53581b1. Accessed 15 February 2026.

[151] METRIN-KG example query-18. https://kg.earthmetabolome.org/metrin/18. Accessed 15 February 2026.

[152] METRIN-KG example query-18 versioned. GitHub. https://kg.earthmetabolome.org/metrin/18/v/53581b1. Accessed 15 February 2026.

[153] METRIN-KG example query-22. https://kg.earthmetabolome.org/metrin/22. Accessed 15 February 2022.

[154] METRIN-KG example query-22 versioned. GitHub. https://kg.earthmetabolome.org/metrin/22/v/53581b1. Accessed 15 February 2026.

[155] METRIN-KG example query-23. https://kg.earthmetabolome.org/metrin/23. Accessed 15 February 2026.

[156] METRIN-KG example query-23 versioned. https://kg.earthmetabolome.org/metrin/23/v/53581b1. Accessed 15 February 2026.

[157] METRIN-KG example query-16. https://kg.earthmetabolome.org/metrin/16. Accessed 15 February 2026.

[158] METRIN-KG example query-16 versioned. GitHub. https://kg.earthmetabolome.org/metrin/16/v/53581b1. Accessed 15 February 2026.

[159] METRIN-KG example query-17. https://kg.earthmetabolome.org/metrin/17. Accessed 15 February 2026.

[160] METRIN-KG example query-17 versioned. GitHub. https://kg.earthmetabolome.org/metrin/17/v/53581b1. Accessed 15 February 2026.

[161] METRIN-KG example query-19. https://kg.earthmetabolome.org/metrin/19. Accessed 15 February 2026.

[162] METRIN-KG example query-19 versioned. GitHub. https://kg.earthmetabolome.org/metrin/19/v/53581b1. Accessed 15 February 2026.

[163] METRIN-KG example query-21. https://kg.earthmetabolome.org/metrin/21. Accessed 15 February 2026.

[164] METRIN-KG example query-21 versioned. GitHub. https://kg.earthmetabolome.org/metrin/21/v/53581b1. Accessed 23 August 2025.

[165] Bolleman J, Emonet V, Altenhoff A et al. A large collection of bioinformatics question-query pairs over federated knowledge graphs: methodology and applications. arXiv. 2024. 10.48550/arXiv.2410.06010. Accessed 23 August 2025. PMC12083453 40378136

[166] Emonet V, Sima A-C, de FTM. A user-friendly SPARQL query editor powered by lightweight metadata. arXiv. 2025. 10.48550/arXiv.2503.02688. Accessed 11 June 2025.

[167] Contribute queries to METRIN-KG. https://github.com/earth-metabolome-initiative/metrin-kg/wiki/Contribute-queries-to-METRIN%E2%80%90KG. Accessed 23 August 2025.

[168] Jupp S, Malone J, Bolleman J et al. The EBI RDF platform: linked open data for the life sciences. Bioinformatics. 2014;30:1338–39. 10.1093/bioinformatics/btt765. 24413672 PMC3998127

[169] Emonet V, Bolleman J, Duvaud S et al. LLM-based SPARQL query generation from natural language over federated knowledge graphs. arXiv. 2025. 10.48550/arXiv.2410.06062. Accessed 11 June 2025.

[170] ExpasyGPT for METRIN-KG . GitHub. https://github.com/earth-metabolome-initiative/metrin-kg/wiki/ExpasyGPT-for-METRIN%E2%80%90KG. Accessed 23 August 2025.

[171] Calleja J A, Domènech G, Sáez L et al. Extinction risk of threatened and non-threatened mosses: reproductive and ecological patterns. Global Ecol Conserv. 2022;38:e02254. 10.1016/j.gecco.2022.e02254.

[172] Gürlek S, Araújo A C, Brummitt N. Predicting the threat status of mosses using functional traits. Plants. 2024;13:2019. 10.3390/plants13152019. 39124136 PMC11314510

[173] Junaedi D I, Nasution T, Putri D M et al. Threatened exotic species of botanical gardens: application of trait-based naturalized species risk scoring assessment. S Afr J Bot. 2023;152:321–31. 10.1016/j.sajb.2022.11.046.

[174] Álvarez-Yépiz J C, Búrquez A, Martínez-Yrízar A et al. A trait-based approach to the conservation of threatened plant species. Oryx. 2019;53:429–35. 10.1017/S003060531800087X.

[175] Hill J L, Grisnik M, Hanscom R J et al. The past, present, and future of predator–prey interactions in a warming world: using species distribution modeling to forecast ectotherm–endotherm niche overlap. Ecol Evol. 2024;14:e11067. 10.1002/ece3.11067. 38435021 PMC10905248

[176] Stelling-Wood T P, Poore AGB, Hughes A R et al. Habitat traits and predation interact to drive abundance and body size patterns in associated fauna. Ecol Evol. 2023;13:e10771. 10.1002/ece3.10771. 38053789 PMC10694384

[177] Ray K, Basak S K, Giri C K et al. Ecological restoration at pilot-scale employing site-specific rationales for small-patch degraded mangroves in Indian Sundarbans. Sci Rep. 2024;14:12952. 10.1038/s41598-024-63281-8. 38839775 PMC11153218

[178] Mendes S B, Nogales M, Vargas P et al. Climb forest, climb: diverse disperser communities are key to assist plants tracking climate change on altitudinal gradients. New Phytol. 2025;245:1315–29. 10.1111/nph.20300. 39621546 PMC11711941

[179] Flickinger H D, Dukes J S. A review of theory: comparing invasion ecology and climate change-induced range shifting. Global Change Biol. 2024;30:e17612. 10.1111/gcb.17612. PMC11647056 39676232

[180] Wang X, Cao Y, Jin Y et al. Ecophysiological trade-off strategies of three gramineous crops in response to root extracts of Phytolacca americana. Plants. 2024;13:3026. 10.3390/plants13213026. 39519945 PMC11548188

[181] De La Peña R, Sattely E S. Rerouting plant terpene biosynthesis enables momilactone pathway elucidation. Nat Chem Biol. 2021;17:205–12. 10.1038/s41589-020-00669-3. 33106662 PMC7990393

[182] Knoch E, Kovács J, Deiber S et al. Transcriptional response of a target plant to benzoxazinoid and diterpene allelochemicals highlights commonalities in detoxification. BMC Plant Biol. 2022;22:402. 10.1186/s12870-022-03780-w. 35974304 PMC9382751

[183] Lu X, Zhang J, Brown B et al. Inferring roles in defense from metabolic allocation of rice diterpenoids. Plant Cell. 2018;30:1119–31. 10.1105/tpc.18.00205. 29691314 PMC6002189

[184] Zhou S, Zhang R, Wang Q et al. OsbHLH5 Synergically regulates phenolamide and diterpenoid phytoalexins involved in the defense of rice against pathogens. Int J Mol Sci. 2024;25:12152. 10.3390/ijms252212152. 39596224 PMC11595221

[185] Vela F, Anese S, Varela R M et al. Bioactive diterpenes from the Brazilian native plant (Moquiniastrum pulchrum) and their application in weed control. Molecules. 2021;26:4632. 10.3390/molecules26154632. 34361785 PMC8348793

[186] Maraia H, Charles-Dominique T, Tomlinson K W et al. Substantial insect herbivory in a South African Savanna-forest mosaic: a neglected topic. Ecol Evol. 2024;14:e70466. 10.1002/ece3.70466. 39524307 PMC11549574

[187] Zhao Y, Hu J, Zhou Z et al. Biofortified rice provides rich sakuranetin in endosperm. Rice. 2024;17:19. 10.1186/s12284-024-00697-w. 38430431 PMC10908774

[188] Khatibi SMH, Dimaano N G, Veliz E et al. Exploring and exploiting the rice phytobiome to tackle climate change challenges. Plant Commun. 2024;5:101078. 10.1016/j.xplc.2024.101078. 39233440 PMC11671768

[189] Bian S, Li Z, Song S et al. Enhancing crop resilience: insights from labdane-related diterpenoid phytoalexin research in rice (Oryza sativa L.). Curr Iss Mol Biol. 2024;46:10677–95. 10.3390/cimb46090634. PMC11430374 39329985

[190] Wang X, Li X, Zhao W et al. Current views of drought research: experimental methods, adaptation mechanisms and regulatory strategies. Front Plant Sci. 2024;15:1371895. 10.3389/fpls.2024.1371895. 38638344 PMC11024477

[191] Yang H, Ji S, Wu D et al. Effects of root–root interactions on the physiological characteristics of haloxylon ammodendron seedlings. Plants. 2024;13:683. 10.3390/plants13050683. 38475528 PMC10934858

[192] Shi Y, He Y, Zheng Y et al. Characteristics of the phyllosphere microbial community and its relationship with major aroma precursors during the tobacco maturation process. Front Plant Sci. 2024;15:1346154. 10.3389/fpls.2024.1346154. 38799095 PMC11116568

[193] Chain F E, Romano E, Leyton P et al. Vibrational and structural study of onopordopicrin based on the FTIR spectrum and DFT calculations. Spectrochim Acta A. 2015;150:381–89. 10.1016/j.saa.2015.05.072. 26057092

[194] Suzuki M, Iwasaki A, Suenaga K et al. Phytotoxic activity of crop residues from Burdock and an active substance. J Environ Sci Health B. 2019;54:877–82. 10.1080/03601234.2019.1636600. 31271331

[195] El Khatib N, Morel S, Hugon G et al. Identification of a sesquiterpene lactone from arctium lappa leaves with antioxidant activity in primary Human muscle cells. Molecules. 2021;26:1328. 10.3390/molecules26051328. 33801315 PMC7958318

[196] Zhang J, Zheng Z-Q, Xu Q et al. Onopordopicrin from the new genus Shangwua as a novel thioredoxin reductase inhibitor to induce oxidative stress-mediated tumor cell apoptosis. J Enzyme Inhib Med Chem. 2021;36:790–801. 10.1080/14756366.2021.1899169. 33733960 PMC7993383

[197] Maeta A, Okamoto Y, Ishikawa H et al. Japanese leaf burdock extract inhibits adipocyte differentiation in 3T3-L1 cells. Plant Foods Hum Nutr. 2025;80:32. 10.1007/s11130-024-01257-9. 39777584

[198] Jalloh A A, Khamis F M, Yusuf A A et al. Long-term push–pull cropping system shifts soil and maize-root microbiome diversity paving way to resilient farming system. BMC Microbiol. 2024;24:92. 10.1186/s12866-024-03238-z. 38500045 PMC10946131

[199] Czarnobai De Jorge B, Koßmann A, Hummel H E et al. Evaluation of a push-and-pull strategy using volatiles of host and non-host plants for the management of pear psyllids in organic farming. Front Plant Sci. 2024;15:1375495. 10.3389/fpls.2024.1375495. 38841281 PMC11150531

[200] Khan Z R, Chiliswa P, Ampong-Nyarko K et al. Utilisation of wild gramineous plants for management of cereal stemborers in Africa. Int J Trop Insect Sci. 1997;17:143–50. 10.1017/S1742758400022268.

[201] Midega CAO, Wasonga C J, Hooper A M et al. Drought-tolerant Desmodium species effectively suppress parasitic striga weed and improve cereal grain yields in western Kenya. Crop Prot. 2017;98:94–101. 10.1016/j.cropro.2017.03.018. 28775391 PMC5465939

[202] Khan Z R, Midega CAO, Amudavi D M et al. On-farm evaluation of the ‘push–pull’ technology for the control of stemborers and striga weed on maize in western Kenya. Field Crops Res. 2008;106:224–33. 10.1016/j.fcr.2007.12.002.

[203] Hooper A M, Caulfield J C, Hao B et al. Isolation and identification of Desmodium root exudates from drought tolerant species used as intercrops against Striga hermonthica. Phytochemistry. 2015;117:380–387. 10.1016/j.phytochem.2015.06.026. 26164239 PMC4560159

[204] Reich P B, Walters M B, Ellsworth D S. From tropics to tundra: global convergence in plant functioning. Proc Natl Acad Sci USA. 1997;94:13730–34. 10.1073/pnas.94.25.13730. 9391094 PMC28374

[205] Tandon D, Mendes De Farias T, Allard P-M et al. METRIN-KG: a knowledge graph integrating plant metabolites, traits and biotic interactions (Version v1.0.2). Zenodo. 10.5281/zenodo.18684960. Accessed 18 February 2026. PMC13296791 42053508

