# Supplementary material for: METRIN-KG: A knowledge graph integrating plant metabolites, traits, and biotic interactions
Source: Gigascience. 2026 Apr 29;15:giag051. doi: 10.1093/gigascience/giag051 (PMC13296791; doi:10.1093/gigascience/giag051)
Supplement: giag051_Authors_Response_To_Reviewer_Comments_original_submission [file giag051_authors_response_to_reviewer_comments_original_submission.pdf]

Author's Response To Reviewer Comments

Dear Editor and Reviewers,

We appreciate the thoughtful and constructive feedback provided by the reviewers, as well as the editors' consideration of our manuscript for publication in GigaScience. In response to the valuable comments and suggestions, we have carefully revised the manuscript to address all points raised during the review process.
We have also added the RRIDs and biotools identifiers in the source code availability section.
Below, we provide a point-by-point response to each reviewer comment, detailing the specific changes made to the manuscript.

REVIEWER #1:This study makes an important and timely contribution to plant ecology by combining multiple data sources of plant functional traits, metabolites, and interaction partners. Linking different aspects of a plants' phenotype (morphological and physiological traits, as well as metabolite profiles) with their potential ecological functions (biotic interactions) represents a much-needed step forward in both chemical and functional ecology. The effort the authors have put into compiling these datasets and establishing connections across sources is evident and deserves appreciation. The potential applications of this framework are manifold, and the examples provided on how the database can be used to explore research questions through knowledge paths convincingly demonstrate its value.
The Introduction could be strengthened. At present, it feels somewhat long and diffuse in scope, which may make it harder for the reader to quickly grasp the importance of the contribution. Streamlining the text and sharpening the focus on the added value that linking different data sources provides would considerably improve clarity and help the reader more fully appreciate the strength of this novel approach.

AUTHORS
We agree with the reviewer that the original Introduction covered a broad scope that could obscure the core contribution. We have therefore broken the Context into 3 sub-sections:
Interplay of Metabolomes, Traits, and Biotic Interactions
Challenges in Integrating High-Dimensional Data
MEtabolomes, TRaits, and INteractions-Knowledge Graph (METRIN-KG)
We have streamlined the information into these subsections to inform the reader about the need to integrate traits, interactions, and metabolite data as well as the existing limitations in doing so and finally the specific added value of integrating these datasets within a unified framework through METRIN-KG. We have also improved the language of the text for better readability. These changes are reflected in the revised manuscript (Context, pages 5–10).

REVIEWER
Minor comments: It would be helpful to specify more clearly what is meant by the "tremendous chemical diversity" that challenges metabolomic data analysis, and to clarify whether the "limited resources available to manage such complexity" refers to analytical limitations, data availability, or both. Similarly, the phrase "multi-level interaction data also lies locked in the guise of pairwise correlation metrics" could benefit from further explanation. As far as I am aware, the GLOBI database reports individual pairwise observations, which could already be seen as an effective way of documenting such interactions.

AUTHORS
We agree that these phrases were general. To address this, we have implemented the following changes:
We have specified that the phrase ‘tremendous chemical diversity’ refers to the plant metabolites estimated to be between 1.5 and 25.7 million.
We have clarified that ‘limited resources’ refers to ‘analytical limitations (e.g. incomplete spectral libraries, detection limits), suboptimal data availability and cross-links across the three components (plant traits, interactions and metabolites)’. The relevant sentences have been revised accordingly (Context, page 8).
We did not intend to imply a limitation of GloBI’s core design, which indeed provides valuable, observation-

level pairwise interaction records. Rather, our point was that higher-order interaction structures (e.g. multi-trophic or trait-mediated interaction contexts) are often analyzed post hoc using correlation based summaries that abstract away mechanistic or phenotypic information. We have now revised the text to distinguish between the availability of pairwise interaction records and the challenges associated with integrating these data with trait and metabolite information to infer broader ecological patterns (Context, page 9).

REVIEWER #2: The authors present METRIN-KG, a knowledge graph integrating plant metabolomic, trait, and biotic interaction datasets. The work should have substantial value for multiple plant sciences and ecology domains. The overall effort to harmonize disparate resources into an integrated, semantically coherent resource is impressive. The methodology includes a notable pipeline for ontology alignment across multiple sources. However, despite its technical strengths, several issues regarding data accessibility and manuscript structure and clarity should be addressed. The manuscript is highly technical throughout. While this level of precision is exemplary, it may alienate biologically oriented readers, and effort should be made so that the impact and manuscript is clear to a larger audience.

AUTHORS
We thank the reviewer for the helpful comments. We have made improvements to our manuscript. Precise changes we made are explained in the following paragraphs.

REVIEWER
Major comments

1. The online deployment currently presents several problems that must be resolved before publication.

The existence of multiple SPARQL UIs (https://kg.earthmetabolome.org/metrin which redirects to https://qlever.earthmetabolome.org/metrin-kg/ and https://sib-swiss.github.io/sparql-editor/metrin-kg) is not explained, and the redundancy is potentially confusing.
At the time of review, https://qlever.earthmetabolome.org/metrin-kg/ showed expired SSL certificates, making it inaccessible for most users. Automatic certificate renewal (e.g., using certbot) should be implemented.
Attempting to use the SIB UI resulted in an error due to the redirect.
The certificate issue also prevented evaluation of ExpasyGPT querying against METRIN-KG.

AUTHORS
We thank the reviewer for carefully evaluating the online deployment and for identifying important technical issues affecting accessibility and usability. We agree that these problems need to be resolved prior to publication, and we have taken concrete steps to address each point raised.
https://kg.earthmetabolome.org/metrin is a persistent and stable URL to future-proof the potential changes in the underlying SPARQL endpoint (https://qlever.earthmetabolome.org/metrin-kg/). Furthermore, the SIB SPARQL UI uses the Qlever endpoint for METRIN-KG (https://kg.earthmetabolome.org/metrin). However, to avoid any confusion, we have now removed the reference to SIB SPARQL UI from the manuscript, though we intend to keep the URL active for our internal reference. These changes are now reflected in the revised manuscript (Methods pages 21,22; Data re-use and case studies page 28).
The SSL certificate for the Qlever endpoint has now been renewed. The endpoint is currently accessible and was tested across several browsers prior to resubmission.
This issue was caused by an incorrect redirect configuration following the certificate expiration. The redirect has now been corrected, and the SIB SPARQL UI is functional when querying. We have verified successful query execution using example queries provided in the manuscript. As mentioned in (a), we have removed the reference to this URL from the manuscript to avoid confusion.
We acknowledge that the SSL certificate issue prevented proper evaluation of ExpasyGPT integration at the

time of review. Following certificate renewal and endpoint stabilization, we have re-tested ExpasyGPT querying against METRIN-KG and confirmed that queries execute as intended.

REVIEWER
2. Usability represents a significant barrier. Many potential users such as biologists without semantic-web or RDF experience are unlikely to be able to interpret the current figures or formulate SPARQL queries. The manuscript briefly mentions ExpasyGPT, which has strong potential to overcome this barrier by allowing natural language querying. This tool should be emphasized more prominently, potentially with a dedicated subsection and discussion of its role in broadening the resource's accessibility.

AUTHORS
We thank the reviewer for highlighting usability as a critical issue and fully agree that the current interface can present a substantial barrier for users without prior experience in semantic web technologies including SPARQL. However, we believe that improving accessibility for biologists and ecologists without RDF expertise is an important goal of this resource and through this manuscript we have made an effort to address this.
In response, we have revised the manuscript to emphasize the role of ExpasyGPT as a natural-language interface to METRIN-KG. We have added a dedicated subsection ('Democratizing METRIN-KG access: querying in natural language') describing how ExpasyGPT enables users to formulate biologically meaningful queries using plain language, thereby lowering the entry barrier for non-technical users. This subsection also clarifies the types of questions that can be addressed (Table 2) and refers to a tutorial that can be used by non-technical users to use ExpasyGPT (Data re-use and case studies, pages 29,30).

REVIEWER
3. To fully demonstrate the relevance of METRIN-KG, the use-case section would benefit from quantitative summaries, visualizations (e.g., distributions, network visualizations), and biological interpretations of query results.

AUTHORS
We have added quantitative summaries and visualisations for all case studies in the manuscript (Data re-use and case studies, pages 31-37). Given the nature of this manuscript (GigaScience Data Note as opposed to a Research Article), we felt that a complete biological interpretation is out of scope of this work. We acknowledge that the relevance of METRIN-KG will benefit from such biological interpretation and we plan to conduct a study for this purpose in a separate work.

REVIEWER
4. The overall manuscript organisation would benefit from restructuring to improve readability and better expose the impact of the work done.

- The methodological content currently in "Mapping of TRY data" and "Mapping of GloBI data" should be moved to the Methods section, while the quantitative outputs (e.g., numbers of records) should be moved into a dedicated Results section.

- The current "Data re-use and case studies" section could be reorganised into Results and Discussion sections,

- Results include: description of outputs from the methodological steps (e.g. the ontology, the successfulness of the mapping process, the size of the final knowledge graph/number of triples, and other relevant metrics; the user interface, including being able to share and add example questions and write NL questions via ExpasyGPT; examples of SPARQL queries and case studies.

- Discussion includes: Reuse potential; case-study interpretations or impact; future directions including planned expansion and enhancing of ontological structure, etc.

AUTHORS
We thank the reviewer for this thoughtful and detailed suggestion regarding manuscript organization and for outlining an alternative structure that would be appropriate for a conventional research article. However, we have submitted this manuscript as a GigaScience Data Note (https://academic.oup.com/gigascience/pages/data_note), which follows a defined structure that differs from a conventional research article and does not include separate Results or Discussion sections. The Data Note format is explicitly designed to integrate methodological description, quantitative summaries of the resulting dataset, and reuse examples across sections such as Methods, Data Description, and Data Reuse. In particular, the Data Reuse section is intended to include summaries of case studies, how the resource can be exploited. Accordingly, we have retained the current organization to remain compliant with the GigaScience Data Note guidelines and to reflect the intended purpose of this article type. The subsections 'Mapping of TRY data' and 'Mapping of GloBI data' are intentionally placed under the 'Data Description' section (formerly 'Data Structure') to more clearly convey their role in describing the dataset.

REVIEWER
5. An overview and evaluation of the KG should be provided, for example the number of plant species, and the distributions of connections. Any gaps or any (potentially) biases due to the input data needs to be acknowledged. For example, it is very likely certain species may be under/overrepresented in either metabolome or interaction datasets, or possibly geographic skews could exist.

AUTHORS
We agree that an overview and evaluation of the knowledge graph, including its scale and potential biases, is important. Quantitative summaries of the KG, such as the number of plant species, entities, and connections, are already provided in the 'Data Description' section (pages 22–24). In addition, we now briefly state the biases originating from interaction and metabolite data in the same section (pages 25) .

REVIEWER
6. The ontology is variously referred to as the "Earth Metabolome Ontology", "EMI ontology", and "EMI". Consistent naming should be adopted throughout the manuscript and associated repositories. It is also unclear whether the ontology is a result of this work. As written, the "Ontology" section under "Methods" reads more like a result/description than a methodological step. Clarifying what components are original contributions and presenting them in Results would strengthen the manuscript. Additionally, the phrases "our proposed framework" and "our approach" are ambiguous, do these refer to the ontology itself, the metadata-mapping pipeline, or the overall integration process? Finally, referring to METRIN-KG as a "tutorial to build a knowledge graph" appears to be a bit out of place, given the topic of the manuscript.

AUTHORS
We thank the reviewer for these observations. We have adopted consistent naming throughout the manuscript and associated repositories, using Earth Metabolome Initiative Ontology (EMI Ontology) as the standard designation.
We clarify that the ontology is an original contribution of this work and constitutes a foundational methodological component. The EMI ontology had to be defined prior to the knowledge graph construction in order to formalize concepts, relations, and mappings across heterogeneous data sources; for this reason, it is intentionally presented in the Methods section (along with the context for its complex nature), directly preceding the knowledge graph construction steps. To make this clearer, we now explicitly state which components are original contributions and which are reused.
In particular, we have added a sentence clarifying that, in addition to making several existing ontologies interoperable, we created more than 100 new concepts and relations, identifiable by the https://w3id.org/emi# (emi:) prefix (e.g. emi:Trait), and that all reused and newly introduced terms are fully documented and publicly available (Methods, pages 18, 19).

We have also clarified the previously ambiguous phrases such as ‘our proposed framework’ (now replaced by ‘our proposed ontology’) and ‘our approach’ (now removed).
We have now clarified that the manuscript does not present METRIN-KG itself as a tutorial; rather, it states that code and a tutorial for building the metabolite-specific knowledge graph component using the EMI ontology are available in the EMI GitHub repository (Methods, page 19).
We hope that these clarifications and the current changes to the concerned paragraphs sufficiently address the reviewer’s concern.

REVIEWER
7. Given its potential relevance beyond this project, the authors are strongly encouraged to publish the code for the metadata-mapping pipeline. In addition, the following details would strengthen the methodological rigor:

- Were any acceptability criteria implemented in the automated step, e.g. a minimum Cosine similarity threshold?

- Were the manual corrections systematically documented?

- In how many cases were manual corrections needed?

- Was any evaluation done on the embedding/model version or the source of errors?

AUTHORS
We thank the reviewer for these valuable suggestions. We have added the link to our metadata-mapping code in the 'Metadata mapping' section of the manuscript (Methods page 15).
We have further addressed each point raised:
The automated pipeline selects the ontology term with the highest cosine similarity to each input term. Cosine similarity values range from -1 to 1. While all terms receive a candidate match, only matches with a similarity score ≥ 0.7 were flagged for manual review. This threshold ensures that low-confidence suggestions are carefully examined, while very low-similarity terms are treated as unmatched.
All candidate matches above the threshold were manually reviewed. We have added this detail to the Methods (pages 16,17) .
Manual corrections were systematically documented in the output, including the original input term, suggested match, final corrected match, ontology IRI, and similarity score.This documentation is available at https://github.com/earth-metabolome-initiative/metrin-kg/tree/import-ontology-data/ontology/data.
It is difficult to report a precise number of cases requiring manual correction because the initial inputs were not single standardized terms, but unique raw values extracted from metadata columns such as body part, life stage, and biological sex.
Many of these entries contained compound expressions, abbreviations, counts, symbols, or multiple entities within a single field, rather than one-to-one mappings. For example, body part entries included values such as “2 guts”, “abdominal cavity”, “abo”, “abomasum/si”, and similar variants. Biological sex fields contained symbolic or aggregated values (e.g. “+”, “-”, “?”), numeric summaries (e.g. “10M,9F”, “11 males, 4 females, 2 juveniles”), or multilingual expressions (e.g. “11 machos | 9 hembras”). Life stage fields similarly contained compound or inconsistent representations, such as “adult; adult; egg”, “adult; larvae”, “0, I, II, III”, or mixed capitalization and punctuation.
As part of the manual curation process, such entries were first parsed using delimiters and normalization rules to separate them into individual candidate terms, which were then mapped independently to ontology entities. Following mapping and manual review, only those components that could be unambiguously aligned to a single ontology concept were retained and subsequently applied in the final knowledge graph construction.
Because a single raw metadata value could expand into multiple candidate terms and mappings, manual corrections occurred at the component level rather than the original input level, making a simple count of corrected cases at the input-record level not meaningful. Nonetheless, all corrections were systematically reviewed to ensure semantic accuracy and consistency. These details have now been added to the manuscript (Methods pages 15,16).

We did not conduct a formal evaluation of the embedding model version or systematic analysis of error sources, as this was beyond the scope of the current manuscript. Our focus was on demonstrating the practical application of embedding-based methods for metadata mapping in metabolomics data integration, rather than on model evaluation and optimization. We acknowledge this as an area for future methodological development.

REVIEWER
Minor comments

1. The manuscript would benefit from proofreading for consistent use of Oxford commas (and an "&" instead of "and").
2. Reference 190 contains a typo "GiHub"
3. References should be checked (e.g. citation for [95] references both METRIN-KG Zenodo and GloBI Zenodo)
4. The METRIN-KG Zenodo link in the article is not to the latest version (Version 5)
5. Consider improving the figures, e.g. use of colour to better communicate the content and refining layouts.
6. The authors should deposit a snapshot of the GitHub repository to Zenodo.

AUTHORS
The manuscript has been carefully proofread, and formatting has been standardized to ensure consistent use of Oxford commas and the use of “&” in place of “and” where appropriate.
The typo in Reference 190 (“GiHub”) has been corrected to “GitHub”.
All references have been reviewed and corrected for accuracy.
The METRIN-KG Zenodo link has been updated to point to the latest available release.
We appreciate the reviewer’s suggestion regarding figure presentation. The current figures follow standard schema representations commonly used in the domain, and the existing color schemes were chosen to clearly convey the intended structure and relationships. For this reason, no major changes to the figures were made.
We have added links to Figure-1 to better represent the linked open data concept.
A snapshot of the GitHub repositoryis available on Zenodo and the corresponding citation has been added to the manuscript (Availability of source code, page 39).

REVIEWER
The following are suggestions to the authors, to be followed by their own judgement:

1. Table 1, Figure 3, Figure 4 could be moved to supplementary material to make space for figures for the case studies.
2. The dense in-text list of ontologies in the "Metadata mapping" section could be replaced with a summarized table (e.g. by moving Supplementary Table 2, but including references to the main text).
3. The full SPARQL queries in "Taxonomy mapping" could be moved to supplementary materials, with a high-level description left in the main text.

AUTHORS
We appreciate the reviewer's thoughtful suggestions for restructuring the manuscript. We have considered each of them and have made the following decisions:

We respectfully prefer to maintain the current presentation of these elements. Table 1 provides critical context for interpreting our results, while Figures 3-4 illustrate key concepts that would be difficult to follow if moved to supplementary material.
We have removed the in-text list of ontologies and added references to the Supplementary table 2.
We agree with this suggestion and have created a new Supplementary Data section where the full SPARQL queries are now provided. We have retained a high-level description in the main text as recommended. We note that while these queries include a hash, Wikidata's SPARQL endpoint could change in future. Unlike

the other hashes provided elsewhere in the manuscript - which are diligently linked to the SPARQL endpoint and also have versioned copies available in GitHub - this particular query does not have such archival support. Therefore, we felt it important to preserve the full queries in the supplementary materials rather than relying solely on the hash reference.

We believe the revised version incorporates the reviewers' recommendations and adequately responds to their questions and concerns.

Editorial Manager®

Cookie Preference Center

We use cookies which are necessary to make our site work. We may also use additional cookies to analyze, improve and personalize our content and your digital experience. For more information, see our Cookie Policy.

You may choose not to allow some types of cookies. However, blocking some types may impact your experience of our site and the services we are able to offer. See the different category headings below to find out more or change your settings.

Allow all

Manage Consent Preferences

Strictly Necessary Cookies

Always active

These cookies are necessary for the website to function and cannot be switched off in our systems. They are usually only set in response to actions made by you which amount to a request for services, such as setting your privacy preferences, logging in or filling in forms. You can set your browser to block or alert you about these cookies, but some parts of the site will not then work.

Functional Cookies

These cookies enable the website to provide enhanced functionality and personalisation. They may be set by us or by third party providers whose services we

have added to our pages. If you do not allow these cookies then some or all of these services may not function properly.

Performance Cookies

These cookies allow us to count visits and traffic sources so we can measure and improve the performance of our site. They help us to know which pages are the most and least popular and see how visitors move around the site.

Targeting Cookies

These cookies may be set through our site by our advertising partners. They may be used by those companies to build a profile of your interests and show you relevant adverts on other sites. If you do not allow these cookies, you will experience less targeted advertising.

Cookie List

Clear

Apply Cancel
Consent Leg.Interest

Confirm my choices

Power
