# Supplementary material for: METRIN-KG: A knowledge graph integrating plant metabolites, traits, and biotic interactions
Source: Gigascience. 2026 Apr 29;15:giag051. doi: 10.1093/gigascience/giag051 (PMC13296791; doi:10.1093/gigascience/giag051)
Supplement: giag051_Reviewer_1_Report_original_submission — Reviewer 1 -- 9/29/2025 [file giag051_reviewer_1_report_original_submission.pdf]

Reviewer Report

Title: METRIN-KG: A knowledge graph integrating plant metabolites, traits, and biotic interactions

Version: Original Submission **Date:** 9/29/2025

Reviewer name: Reviewer 1

Reviewer Comments to Author:

This study makes an important and timely contribution to plant ecology by combining multiple data sources of plant functional traits, metabolites, and interaction partners. Linking different aspects of a plants' phenotype (morphological and physiological traits, as well as metabolite profiles) with their potential ecological functions (biotic interactions) represents a much-needed step forward in both chemical and functional ecology. The effort the authors have put into compiling these datasets and establishing connections across sources is evident and deserves appreciation. The potential applications of this framework are manifold, and the examples provided on how the database can be used to explore research questions through knowledge paths convincingly demonstrate its value.

The Introduction could be strengthened. At present, it feels somewhat long and diffuse in scope, which may make it harder for the reader to quickly grasp the importance of the contribution. Streamlining the text and sharpening the focus on the added value that linking different data sources provides would considerably improve clarity and help the reader more fully appreciate the strength of this novel approach.

Minor comments: It would be helpful to specify more clearly what is meant by the "tremendous chemical diversity" that challenges metabolomic data analysis, and to clarify whether the "limited resources available to manage such complexity" refers to analytical limitations, data availability, or both. Similarly, the phrase "multi-level interaction data also lies locked in the guise of pairwise correlation metrics" could benefit from further explanation. As far as I am aware, the GLOBI database reports individual pairwise observations, which could already be seen as an effective way of documenting such interactions.

Methods

Are the methods appropriate to the aims of the study, are they well described, and are necessary controls included? Choose an item.

Conclusions

Are the conclusions adequately supported by the data shown? Choose an item.

Reporting Standards

Does the manuscript adhere to the journal's guidelines on minimum standards of reporting? Choose an item.

Choose an item.

Statistics

Are you able to assess all statistics in the manuscript, including the appropriateness of statistical tests used? Choose an item.

Quality of Written English

Please indicate the quality of language in the manuscript: Choose an item.

Declaration of Competing Interests

Please complete a declaration of competing interests, considering the following questions:

- Have you in the past five years received reimbursements, fees, funding, or salary from an organisation that may in any way gain or lose financially from the publication of this manuscript, either now or in the future?
- Do you hold any stocks or shares in an organisation that may in any way gain or lose financially from the publication of this manuscript, either now or in the future?
- Do you hold or are you currently applying for any patents relating to the content of the manuscript?
- Have you received reimbursements, fees, funding, or salary from an organization that holds or has applied for patents relating to the content of the manuscript?
- Do you have any other financial competing interests?
- Do you have any non-financial competing interests in relation to this paper?

If you can answer no to all of the above, write 'I declare that I have no competing interests' below. If your reply is yes to any, please give details below.

I declare that I have no competing interests

I agree to the open peer review policy of the journal. I understand that my name will be included on my report to the authors and, if the manuscript is accepted for publication, my named report including any attachments I upload will be posted on the website along with the authors' responses. I agree for my report to be made available under an Open Access Creative Commons CC-BY license (http://creativecommons.org/licenses/by/4.0/). I understand that any comments which I do not wish to be included in my named report can be included as confidential comments to the editors, which will not be published.

Choose an item.

To further support our reviewers, we have joined with Publons, where you can gain additional credit to further highlight your hard work (see: https://publons.com/journal/530/gigascience). On publication of this paper, your review will be automatically added to Publons, you can then choose whether or not to claim your Publons credit. I understand this statement.

Yes Choose an item.
