# Supplementary material for: METRIN-KG: A knowledge graph integrating plant metabolites, traits, and biotic interactions
Source: Gigascience. 2026 Apr 29;15:giag051. doi: 10.1093/gigascience/giag051 (PMC13296791; doi:10.1093/gigascience/giag051)
Supplement: giag051_Reviewer_2_Report_original_submission — Reviewer 2 -- 11/19/2025 [file giag051_reviewer_2_report_original_submission.pdf]

Reviewer Report

Title: METRIN-KG: A knowledge graph integrating plant metabolites, traits, and biotic interactions

Version: Original Submission **Date:** 11/19/2025

Reviewer name: Reviewer 2

Reviewer Comments to Author:

The authors present METRIN-KG, a knowledge graph integrating plant metabolomic, trait, and biotic interaction datasets. The work should have substantial value for multiple plant sciences and ecology domains. The overall effort to harmonize disparate resources into an integrated, semantically coherent resource is impressive. The methodology includes a notable pipeline for ontology alignment across multiple sources. However, despite its technical strengths, several issues regarding data accessibility and manuscript structure and clarity should be addressed. The manuscript is highly technical throughout. While this level of precision is exemplary, it may alienate biologically oriented readers, and effort should be made so that the impact and manuscript is clear to a larger audience.

Major comments

1. The online deployment currently presents several problems that must be resolved before publication.

- The existence of multiple SPARQL UIs (https://kg.earthmetabolome.org/metrin which redirects to https://qlever.earthmetabolome.org/metrin-kg/ and https://sib-swiss.github.io/sparql-editor/metrin-kg) is not explained, and the redundancy is potentially confusing.
- At the time of review, https://qlever.earthmetabolome.org/metrin-kg/ showed expired SSL certificates, making it inaccessible for most users. Automatic certificate renewal (e.g., using certbot) should be implemented.
- Attempting to use the SIB UI resulted in an error due to the redirect.
- The certificate issue also prevented evaluation of ExpasyGPT querying against METRIN-KG.

2. Usability represents a significant barrier. Many potential users such as biologists without semantic-web or RDF experience are unlikely to be able to interpret the current figures or formulate SPARQL queries. The manuscript briefly mentions ExpasyGPT, which has strong potential to overcome this barrier by allowing natural language querying. This tool should be emphasized more prominently, potentially with a dedicated subsection and discussion of its role in broadening the resource's accessibility.

3. To fully demonstrate the relevance of METRIN-KG, the use-case section would benefit from quantitative summaries, visualizations (e.g., distributions, network visualizations), and biological interpretations of query results.

4. The overall manuscript organisation would benefit from restructuring to improve readability and better expose the impact of the work done.

- The methodological content currently in "Mapping of TRY data" and "Mapping of GloBI data" should be moved to the Methods section, while the quantitative outputs (e.g., numbers of records) should be moved into a dedicated Results section.
- The current "Data re-use and case studies" section could be reorganised into Results and Discussion

sections,
- Results include: description of outputs from the methodological steps (e.g. the ontology, the successfulness of the mapping process, the size of the final knowledge graph/number of triples, and other relevant metrics; the user interface, including being able to share and add example questions and write NL questions via ExpasyGPT; examples of SPARQL queries and case studies.
- Discussion includes: Reuse potential; case-study interpretations or impact; future directions including planned expansion and enhancing of ontological structure, etc.

5. An overview and evaluation of the KG should be provided, for example the number of plant species, and the distributions of connections. Any gaps or any (potentially) biases due to the input data needs to be acknowledged. For example, it is very likely certain species may be under/overrepresented in either metabolome or interaction datasets, or possibly geographic skews could exist.
6. The ontology is variously referred to as the "Earth Metabolome Ontology", "EMI ontology", and "EMI". Consistent naming should be adopted throughout the manuscript and associated repositories. It is also unclear whether the ontology is a result of this work. As written, the "Ontology" section under "Methods" reads more like a result/description than a methodological step. Clarifying what components are original contributions and presenting them in Results would strengthen the manuscript. Additionally, the phrases "our proposed framework" and "our approach" are ambiguous, do these refer to the ontology itself, the metadata-mapping pipeline, or the overall integration process? Finally, referring to METRIN-KG as a "tutorial to build a knowledge graph" appears to be a bit out of place, given the topic of the manuscript.
7. Given its potential relevance beyond this project, the authors are strongly encouraged to publish the code for the metadata-mapping pipeline. In addition, the following details would strengthen the methodological rigor:
- Were any acceptability criteria implemented in the automated step, e.g. a minimum Cosine similarity threshold?
- Were the manual corrections systematically documented?
- In how many cases were manual corrections needed?
- Was any evaluation done on the embedding/model version or the source of errors?

Minor comments

1. The manuscript would benefit from proofreading for consistent use of Oxford commas (and an "&" instead of "and").
2. Reference 190 contains a typo "GiHub"
3. References should be checked (e.g. citation for [95] references both METRIN-KG Zenodo and GloBI Zenodo)
4. The METRIN-KG Zenodo link in the article is not to the latest version (Version 5)
5. Consider improving the figures, e.g. use of colour to better communicate the content and refining layouts.
6. The authors should deposit a snapshot of the GitHub repository to Zenodo.

The following are suggestions to the authors, to be followed by their own judgement:

1. Table 1, Figure 3, Figure 4 could be moved to supplementary material to make space for figures for the case studies.
2. The dense in-text list of ontologies in the "Metadata mapping" section could be replaced with a

summarized table (e.g. by moving Supplementary Table 2, but including references to the main text).
3. The full SPARQL queries in "Taxonomy mapping" could be moved to supplementary materials, with a high-level description left in the main text.

Methods

Are the methods appropriate to the aims of the study, are they well described, and are necessary controls included? Choose an item.

Conclusions

Are the conclusions adequately supported by the data shown? Choose an item.

Reporting Standards

Does the manuscript adhere to the journal's guidelines on [minimum standards of reporting?]() Choose an item.

Choose an item.

Statistics

Are you able to assess all statistics in the manuscript, including the appropriateness of statistical tests used? Choose an item.

Quality of Written English

Please indicate the quality of language in the manuscript: Choose an item.

Declaration of Competing Interests

Please complete a declaration of competing interests, considering the following questions:

- Have you in the past five years received reimbursements, fees, funding, or salary from an organisation that may in any way gain or lose financially from the publication of this manuscript, either now or in the future?
- Do you hold any stocks or shares in an organisation that may in any way gain or lose financially from the publication of this manuscript, either now or in the future?
- Do you hold or are you currently applying for any patents relating to the content of the manuscript?
- Have you received reimbursements, fees, funding, or salary from an organization that holds or has applied for patents relating to the content of the manuscript?
- Do you have any other financial competing interests?
- Do you have any non-financial competing interests in relation to this paper?

If you can answer no to all of the above, write 'I declare that I have no competing interests' below. If your reply is yes to any, please give details below.

I declare that I have no competing interests

I agree to the open peer review policy of the journal. I understand that my name will be included on my report to the authors and, if the manuscript is accepted for publication, my named report including any attachments I upload will be posted on the website along with the authors' responses. I agree for my report to be made available under an Open Access Creative Commons CC-BY license (http://creativecommons.org/licenses/by/4.0/). I understand that any comments which I do not wish to be included in my named report can be included as confidential comments to the editors, which will not be published.

Choose an item.

To further support our reviewers, we have joined with Publons, where you can gain additional credit to further highlight your hard work (see: https://publons.com/journal/530/gigascience). On publication of this paper, your review will be automatically added to Publons, you can then choose whether or not to claim your Publons credit. I understand this statement.

Yes Choose an item.
